# A Phase II, Open-label, Multicenter Study of the Safety and Efficacy of TAK-007 in Adult Patients with Relapsed/Refractory B-cell Non–Hodgkin Lymphoma

**DOI:** 10.1158/2643-3230.BCD-25-0323

**Published:** 2026-04-17

**Authors:** Justin M. Darrah, Indumathy Varadarajan, Amitkumar Mehta, Jennifer N. Saultz, Matthew S. McKinney, Monalisa Ghosh, Usama Gergis, Andrew Krueger, Kevin J. Galinsky, Girish Bende, Ajeetha Ramachandran, Varsha Sundaresan, Siddha Kasar, Bradley Hupf, Sharon Chen, Leopold Sellner, Reem Karmali

**Affiliations:** 1 Internal Medicine/Division of Hematology and Cellular Therapy, Cedars-Sinai Medical Center, Los Angeles, California.; 2 Hematology Oncology, University of Virginia, Charlottesville, Virginia.; 3 Medicine, University of Alabama at Birmingham, Birmingham, Alabama.; 4 Medicine, Division of Hematology/Medical Oncology, Oregon Health & Science University, Portland, Oregon.; 5 Division of Hematologic Malignancies, Department of Medicine, Duke Cancer Institute, Durham, North Carolina.; 6 Department of Internal Medicine, University of Michigan, Ann Arbor, Michigan.; 7 Oncology, Thomas Jefferson University, Philadelphia, Pennsylvania.; 8 Oncology Therapeutic Area Unit, Takeda Development Center Americas, Inc. (TDCA), Cambridge, Massachusetts.; 9 Statistical and Quantitative Sciences, Takeda Development Center Americas, Inc. (TDCA), Cambridge, Massachusetts.; 10 Division of Hematology and Oncology, Northwestern Memorial Hospital, Chicago, Illinois.

## Abstract

In relapsed/refractory (r/r) large B-cell lymphoma (LBCL), chimeric antigen receptor (CAR) natural killer (NK) cell therapies offer potential advantages over CAR T-cell therapies, including off-the-shelf availability and scalable manufacturing. We performed a phase II study of single-dose TAK-007, a cryopreserved, cord blood-derived, off-the-shelf CD19 CAR NK-cell therapy, in 26 heavily pretreated patients with r/r B-cell non–Hodgkin lymphoma (NHL). Adverse events were manageable, with limited grade 1 to 2 cytokine release syndrome (11.5%). The overall response rate with TAK-007 800 × 10^6^ CD19 CAR+ viable NK cells (*n* = 14) was 60.9% [95% confidence interval (CI), 38.5–80.3]. The median progression-free survival was 2 months (95% CI, 0.99–3.58) in LBCL (*n* = 10) and 5.6 months (95% CI, 1.05–8.57) in indolent NHL (*n* = 7) expansion cohorts. Deep molecular responses, including circulating tumor DNA–negative responses, were achieved. Not all TAK-007 products elicited clinical responses; single-cell RNA sequencing showed NK proliferating and NK-CD56^dim^ cell enrichment in TAK-007 products eliciting/not eliciting responses, respectively, which could inform product selection.

**Significance:**

Patients with r/r B-NHL treated with one dose of cryopreserved 800× 10^6^ TAK-007 (CD19 CAR NK cells) had an overall response rate of 60.9%. The safety profile was favorable. Deep molecular responses were achieved; cell subsets that could improve outcomes and inform future product selection were identified.

*See related commentary by Duléry et al., p. 856*

## Teaser

TAK-007, a cryopreserved off-the-shelf CD19 CAR–NK-cell therapy, showed manageable safety and clinical activity in relapsed/refractory B-cell NHL, with product-associated cellular features linked to response.

## Introduction

Chimeric antigen receptor (CAR) T-cell therapies have demonstrated sustainable and curative potential in the treatment of hematologic malignancies, particularly leukemia and lymphoma, with several CD19 CAR T-cell therapies approved to date (1–4). Administration of CAR T-cell therapies is often limited to academic healthcare facilities, imposing geographical and financial barriers (1–6). Their autologous nature results in a lengthy and complex production process, treatment delays, and the need for interim bridging therapies (7). Additionally, CAR T-cell therapies have a risk of critical life-threatening toxicities, such as serious cytokine release syndrome (CRS) and immune effector cell-associated neurotoxicity syndrome (ICANS; ref. 8). Allogeneic CAR T therapies may offer potential advantages such as off-the-shelf availability and scalability but present challenges such as risk of graft-versus-host disease (GvHD) and allo-rejection (9). Thus, there is still a need for additional therapies that are clinically effective and safe and have a fast, cost-effective, and high-volume manufacturing process for patients who experience disease progression on, or are not eligible for, autologous CAR T-cell therapies (10).

CAR-natural killer (NK) cells have the potential to overcome these limitations. They can be generated from a variety of sources, such as cord blood (CB), peripheral blood, or induced pluripotent stem cells, and may have a lower risk of rejection than traditional CAR T-cell therapies (11–13). CAR-NK cells may be administered without recipient human leukocyte antigen (HLA) matching, thereby eliminating the need to produce an individualized CAR product (14).

CAR-NK cells derived from CB and genetically modified with an anti-CD19 CAR, the CD28 costimulatory and CD3 zeta signal activation domains, and a soluble interleukin 15 (*IL15*) gene to prolong persistence of NK cells in the patient have been previously investigated in a phase II/II study (NCT03056339; refs. 12, 15). In this trial, CAR-NK cells were expanded for 14 days and infused fresh into adult patients with relapsed/refractory (r/r) CD19^+^ B-cell malignancies. This study demonstrated an overall response rate (ORR) of 49% with durable responses observed in some patients and a 1-year progression-free survival (PFS) rate of approximately 32%. Notably, patients who received CAR-NK cells derived from an “optimal” CB unit, defined as cryopreserved within 24 hours and containing <8 × 10^7^ nucleated red blood cells, achieved a markedly higher 1-year PFS of 68%. Importantly, no significant toxicities such as CRS, ICANS, or GvHD were reported (14, 15).

**Figure 1.**
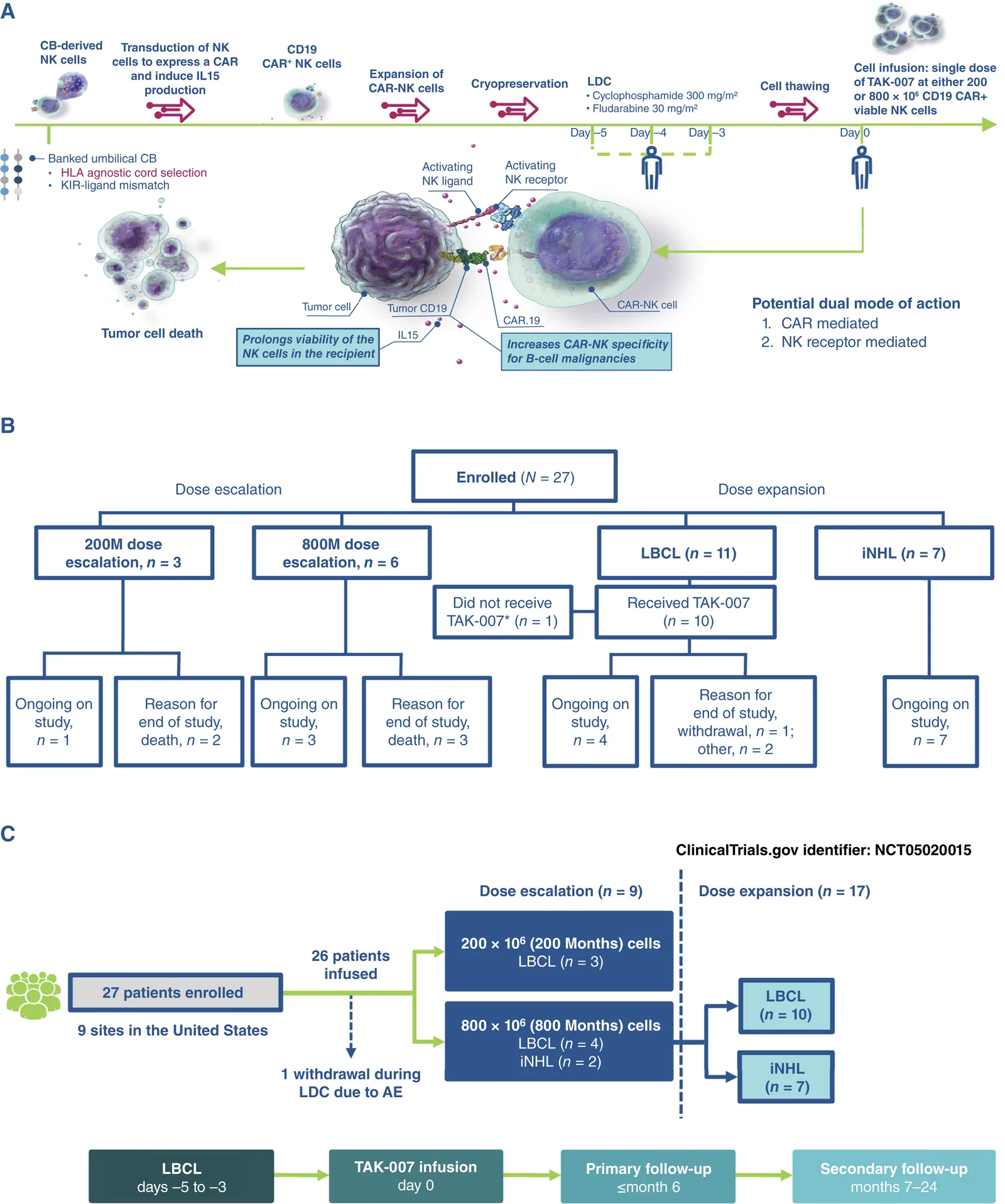
**A,** TAK-007 manufacturing and mechanism of action. **B,** Patient disposition. Patients were ongoing on study (period during which the patient was actively enrolled as per protocol) as of data cutoff of July 1, 2024. **C,** Study design. *Did not receive TAK-007 due to an adverse event; ^†^Cyclophosphomide 300 mg/m^2^ and fludarabine 30 mg/m^2^. AE, adverse event; KIR, killer cell immunoglobulin–like receptor.

TAK-007 is a cryopreserved, allogeneic, CAR-NK cell therapy using the same genetic construct and NK cell source as the aforementioned CD19 CAR-NK cells. For TAK-007, we implemented significant modifications to the manufacturing process. Unlike the original anti-CD19 CAR NK-cell product, which used fresh CD19 CAR-NK cells that were expanded for 14 days only, TAK-007 was expanded to 25 days to achieve higher cell yields, and a cryopreservation protocol was developed enabling the production of a frozen CAR NK-cell product for off-the-shelf availability. These refinements facilitated the use of cryopreserved CAR-NK cells, offering greater flexibility and scalability compared with the use of fresh CAR-NK cells. TAK-007 not only binds to and induces selective cytotoxicity against CD19-expressing cells via CAR-mediated killing mechanisms but also kills CD19-negative tumor cells that express high levels of stress ligands via innate NK receptor–mediated cytotoxicity (Fig. 1A).

In this study, we present data from a phase II study of TAK-007 (NCT05020015) which investigated the safety, efficacy, cellular kinetics (CK), and pharmacodynamics in adult patients with r/r B-cell non–Hodgkin lymphoma (NHL), including large B-cell lymphoma (LBCL) and indolent NHL (iNHL).

## Results

### Patient Baseline Demographics and Disease Characteristics

Overall, 26 patients were treated with a single dose of TAK-007, an off-the-shelf CD19 CAR NK-cell therapy expressing IL15, in outpatient (*n* = 13) or inpatient settings (*n* = 13). Inpatient TAK-007 administration was due to logistic considerations and not due to toxicity concerns. In the dose escalation cohorts, three patients received TAK-007 200 × 10^6^ CD19 CAR+ viable NK cells (200M), and six received TAK-007 800 × 10^6^ CD19 CAR+ viable NK cells (800M). The recommended phase II dose (RP2D) for the dose expansion cohort was TAK-007 800M; 10 and 7 patients received 800M in the LBCL and iNHL expansion cohorts, respectively (Fig. 1B and C). As of the data cutoff of July 1, 2024, median follow-up was 5.95 (range, 1.6 to 18) months and 7.87 (range, 1.6 to 18.7) months in the 200M and 800M dose escalation cohorts, respectively. The median follow-up was 2.50 (range, 0.3 to 7.4) months and 11.93 (range, 6.3 to 12.7) months in the 800M LBCL and iNHL dose expansion cohorts, respectively. All patients were followed for the primary follow-up period of 6 months. Patient demographics and key baseline characteristics are included in Table 1. The representativeness of the study patients is reported in Supplementary Table S1. Patients were heavily pretreated with a median of 5 prior therapies (range, 2 to 11), and those treated at 800M with LBCL and iNHL had a similar number of prior therapies (median 4.5 vs. 5 lines of therapy).

**Table 1.** Baseline patient demographics and disease characteristics in the modified ITT population.

Characteristic	200M escalation cohort (*n* = 3)	800M escalation and expansion cohorts		All patients (*N* = 26)
		LBCL (*n* = 14)	iNHL (*n* = 9)	
Median age, years (range)	61 (59–69)	65 (38–92)	64 (49–83)	64 (38–83)
Male, *n* (%)	2 (66.7)	12 (85.7)	4 (44.4)	18 (69.2)
Race, *n* (%)				
White	3 (100)	11 (78.6)	9 (100)	23 (88.5)
Black or African American	0	1 (7.1)	0	1 (3.8)
Asian	0	1 (7.1)	0	1 (3.8)
Multiple	0	1 (7.1)	0	1 (3.8)
Ethnicity, *n* (%)				
Hispanic or Latino	0	0	1 (11.1)	1 (3.8)
Not Hispanic or Latino	3 (100)	13 (92.9)	8 (88.9)	24 (92.3)
Not reported	0	1 (7.1)	0	1 (3.8)
Baseline ECOG PS				
0	1 (33.3)	2 (14.3)	6 (66.7)	9 (34.6)
1	2 (66.7)	12 (85.7)	3 (33.3)	17 (65.4)
Disease subtype at study entry, *n* (%)				
LBCL NOS	2 (66.7)	6^a^ (42.9)	0	8 (30.8)
LBCL arising from iNHL including FL or MZL	1 (33.3)	6 (42.9)	0	7 (26.9)
HGBL with *MYC*/*BCL2*/*BLC6* rearrangement	0	2 (14.3)	0	2 (7.7)
Follicular lymphoma	0	0	8 (88.9)	8 (30.8)
Splenic MZL	0	0	1 (11.1)	1 (3.8)
Prior number of lines of therapy, median (range)	6 (5–6)	4 (2–11)	5 (2–9)	5 (2–11)
Patients with prior anti-CD19 therapies, *n* (%)	2 (66.7)	7 (50)	2 (22.2)	11 (42.3)
CAR T-cell therapies	2 (66.7)	6 (42.9)	1 (11.1)	9 (34.6)
Response to the last line of therapy, *n* (%)				
Refractory^b^	1 (33.3)	8 (57.1)	2 (22.2)	12 (42.3)
Relapsed^c^	1 (33.3)	5 (35.7)	6 (66.7)	12 (46.2)
Unknown	1 (33.3)	1 (7.1)	1 (11.1)	3 (11.5)
Disease stage at study entry, *n* (%)				
I–II	1 (33.3)	3 (21.4)	0	4 (15.4)
III–IV	2 (66.7)	9 (64.3)	9 (100)	20 (76.9)
Unknown	0	2 (14.3)	0	2 (7.7)
FLIPI score at study entry (FL only)^d^, *n* (%)				
0–1	0	0	0	0
2–3	0	0	5 (62.5)	5 (62.5)
4–5	0	0	3 (37.5)	3 (37.5)
IPI score at study entry (LBCL only)^e^, *n* (%)				
0–1	2 (66.7)	0	0	2 (11.8)
2–3	1 (33.3)	9 (64.3)	0	10 (58.8)
4–5	0	2 (14.3)	0	2 (11.8)
Unknown	0	3 (21.4)	0	3 (17.6)
Molecular classification at study entry (LBCL only)^e^, *n* (%)				
GCB	1 (33.3)	4 (28.6)	0	5 (29.4)
ABC/non-GCB	1 (33.3)	10 (71.4)	0	11 (64.7)
Unknown	1 (33.3)	0	0	1 (5.9)
Bone marrow involvement at study entry				
Yes	0	0	5 (55.6)	5 (19.2)
No	3 (100)	14 (100)	4 (44.4)	21 (80.8)
Unknown	0	0	0	0
Extranodal involvement at initial diagnosis				
Yes	2 (66.7)	12 (85.7)	2 (22.2)	16 (61.5)
No	1 (33.3)	2 (14.3)	7 (77.8)	10 (38.5)
Unknown	0	0	0	0
POD24 (iNHL only)^f^, *n* (%)				
Yes	0	0	6 (66.7)	6 (66.7)
No	0	0	3 (33.3)	3 (33.3)
Unknown	0	0	0	0

Abbreviations: ABC, activated B cell; BCL2, B-cell lymphoma 2; DLBCL, diffuse large B-cell lymphoma; ECOG PS, Eastern Cooperative Oncology Group performance status; FL, follicular lymphoma; FLIPI, Follicular Lymphoma International Prognostic Index; GBC, germinal center B cell; HGBL, high-grade B-cell lymphoma; IPI, International Prognostic Index; MZL, marginal zone lymphoma; NOS, not otherwise specified; POD24, disease progression within 24 months.

a Includes one patient with Epstein–Barr virus–positive DLBCL-NOS.

b Defined as patients who had disease progression as the best response to the last line of therapy or stable disease for <6 months after the last line of therapy.

c Defined as patients who had a PR or CR at the last line of therapy and relapsed prior to the study.

d The percentage is based on the number of patients with FL in the corresponding treatment group at study entry.

e The percentage is based on the number of patients with LBCL in the corresponding treatment group at study entry.

f The percentage is based on the number of patients with iNHL in the corresponding treatment group at study entry. POD24 is defined as disease progression within 24 months after any systemic chemoimmunotherapy or during CD20 maintenance.

In total, 11 (42.3%) patients received prior CD19-targeted therapies, with nine (34.6%) of these patients receiving CAR T-cell therapies. Prior CAR T-cell therapies were received by two patients in the 200M escalation cohort, by six patients in the 800M LBCL escalation and expansion cohort, and by one patient in the iNHL escalation and expansion cohort. In the 200M escalation cohort, the median time from last CAR T-cell therapy to lymphodepletion start was 9.15 months; it was 10.50 and 9.53 months in the LBCL and iNHL 800M dose escalation and expansion cohorts, respectively.

### Drug Product Availability at Study Site

The drug product could be delivered within 2 days and was available within a median of 7 days (range, 2 to 17) at the study site after submission of the ordering request, highlighting the off-the-shelf availability of TAK-007.

### Safety

All 26 patients had treatment-emergent adverse events (TEAE), and hematologic TEAEs were reported in 24 (92.3%) patients (Table 2). In the 200M escalation cohort (*n* = 3), the most frequently reported TEAEs were nausea, anemia, fatigue, neutropenia, hypotension, dehydration, leukopenia, constipation, and hyponatremia (each 66.7%). Among patients with LBCL who received TAK-007 800M (*n* = 14), the most common TEAEs were neutropenia (78.6%), leukopenia (71.4%), anemia (64.3%), nausea and fatigue (each 57.1%), thrombocytopenia (42.9%), and pyrexia (42.9%); among patients with iNHL who received TAK-007 800M (*n* = 9), the most common TEAEs were neutropenia (66.7%), nausea (55.6%), and fatigue (44.4%). Overall, most (77%) of the non-hematological TEAEs were grade 1 to 2.

**Table 2.** TEAEs reported by ≥15% of patients and all corresponding grade ≥3 TEAEs in the safety population^a^.

*n* (%)	TEAE (*N* = 26)	Grade ≥3 TEAE (*N* = 26)
Blood and lymphatic system		
Anemia	14 (53.8)	10 (38.5)
Neutropenia^b^	20 (76.9)	19 (73.1)
Thrombocytopenia^c^	11 (42.3)	10 (38.5)
Lymphocyte count decreased	7 (26.9)	7 (26.9)
Leukopenia^d^	14 (53.8)	13 (50)
Gastrointestinal		
Nausea	15 (57.7)	0
Diarrhea	6 (23.1)	0
Constipation	5 (19.2)	0
Vomiting	5 (19.2)	1 (3.8)
Abdominal pain	4 (15.4)	1 (3.8)
Metabolism and nutrition		
Dehydration	7 (26.9)	1 (3.8)
Hypophosphatemia	6 (23.1)	0
Hypokalemia	6 (23.1)	1 (3.8)
Decreased appetite	5 (19.2)	0
Hyponatremia	4 (15.4)	1 (3.8)
Hypomagnesemia	4 (15.4)	0
Vascular		
Hypotension	8 (30.8)	2 (7.7)
Respiratory, thoracic, and mediastinal		
Dyspnea	6 (23.1)	1 (3.8)
Cough	4 (15.4)	0
Musculoskeletal and connective tissue		
Back pain	4 (15.4)	2 (7.7)
Nervous system		
Dizziness	4 (15.4)	0
Headache	4 (15.4)	1 (3.8)
Cardiac		
Atrial fibrillation	4 (15.4)	1 (3.8)
General and administration site		
Fatigue	14 (53.8)	2 (7.7)
Pyrexia	9 (34.6)	1 (3.8)
Edema peripheral	4 (15.4)	1 (3.8)

a TEAEs are listed regardless of causality.

b Includes neutrophil count decreased.

c Includes platelet count decreased.

d Includes white blood cell count decreased.

In total, grade ≥3 TEAEs were reported in 24 (92.3%) patients: 3 (100%) in the 200M cohort, 5 (83.3%) in the 800M escalation cohort, 10 (100%) in the 800M LBCL expansion cohort, and 6 (85.7%) in the 800M iNHL expansion cohort. In patients with LBCL who received TAK-007 800M, the most commonly reported grade ≥3 TEAEs were neutropenia (78.6%), leukopenia (64.3%), and anemia (50%). Neutropenia (66.7%) was the most common grade ≥3 TEAE in patients with iNHL who received TAK-007 800M. Across all cohorts, 24 (9.6%) grade ≥3 TEAEs were related to both lymphodepleting chemotherapy (LDC) and TAK-007, whereas 109 (45.6%) grade ≥3 TEAEs were related to LDC only and 4 (1.7%) were related to TAK-007 only. Grade ≥3 infections were reported in 8 (30.8%) patients, and 3 of them (11.5%) also reported febrile neutropenia. Overall, 16 patients had serious adverse events (SAE) requiring or prolonging hospitalization, including pyrexia (*n* = 4), febrile neutropenia (*n* = 3), and bacterial sepsis (*n* = 2); one SAE (CRS) was related to TAK-007 and LDC, and seven SAEs across five patients were related to LDC only. Of note, 21 (81%) patients had grade ≥2 cytopenia at baseline.

TEAEs of special clinical interest are shown in Table 3. In total, three (11.5%) patients experienced low-grade CRS considered related to TAK-007 administration (*n* = 1 grade 1 in the 200M cohort, *n* = 1 grade 1 in the 800M LBCL cohort, and *n* = 1 grade 2 in the 800M iNHL cohort). In both the 200M and 800M escalation cohorts, one patient had grade 1 pyrexia. In the iNHL 800M dose expansion cohort, one patient had grade 2 pyrexia, chills, and hypoxia. Of note, the patient with hypoxia already had mild hypoxia at baseline. There were no cases of ICANS related to TAK-007. An infusion-related reaction was observed in two patients in the 800M LBCL expansion cohort, one after fludarabine and one after TAK-007 infusion. There were no reports of GvHD or any dose-limiting toxicities (DLT), and no notable changes in vital signs were observed.

**Table 3.** TEAEs of clinical interest related to TAK-007 in the safety population^a^.

*n* (%)	200M escalation (*n* = 3)	800M escalation and expansion cohorts		All patients (*N* = 26)
		LBCL (*n* = 14)	iNHL (*n* = 9)	
CRS				
Grade 1	1 (33)	1 (7)	0	2 (8)
Grade 2	0	0	1 (11)	1 (4)
Grade ≥3	0	0	0	0
ICANS	0	0	0	0
GvHD	0	0	0	0
Infusion-related reaction	0	1 (7)	0	1 (4)
Grade 1	0	1 (7)	0	1 (4)
Grade 2	0	0	0	0
Grade ≥3	0	0	0	0

Abbreviation: IPI, International Prognostic Index.

a TEAEs are regardless of relationship.

### Efficacy

**Figure 2.**
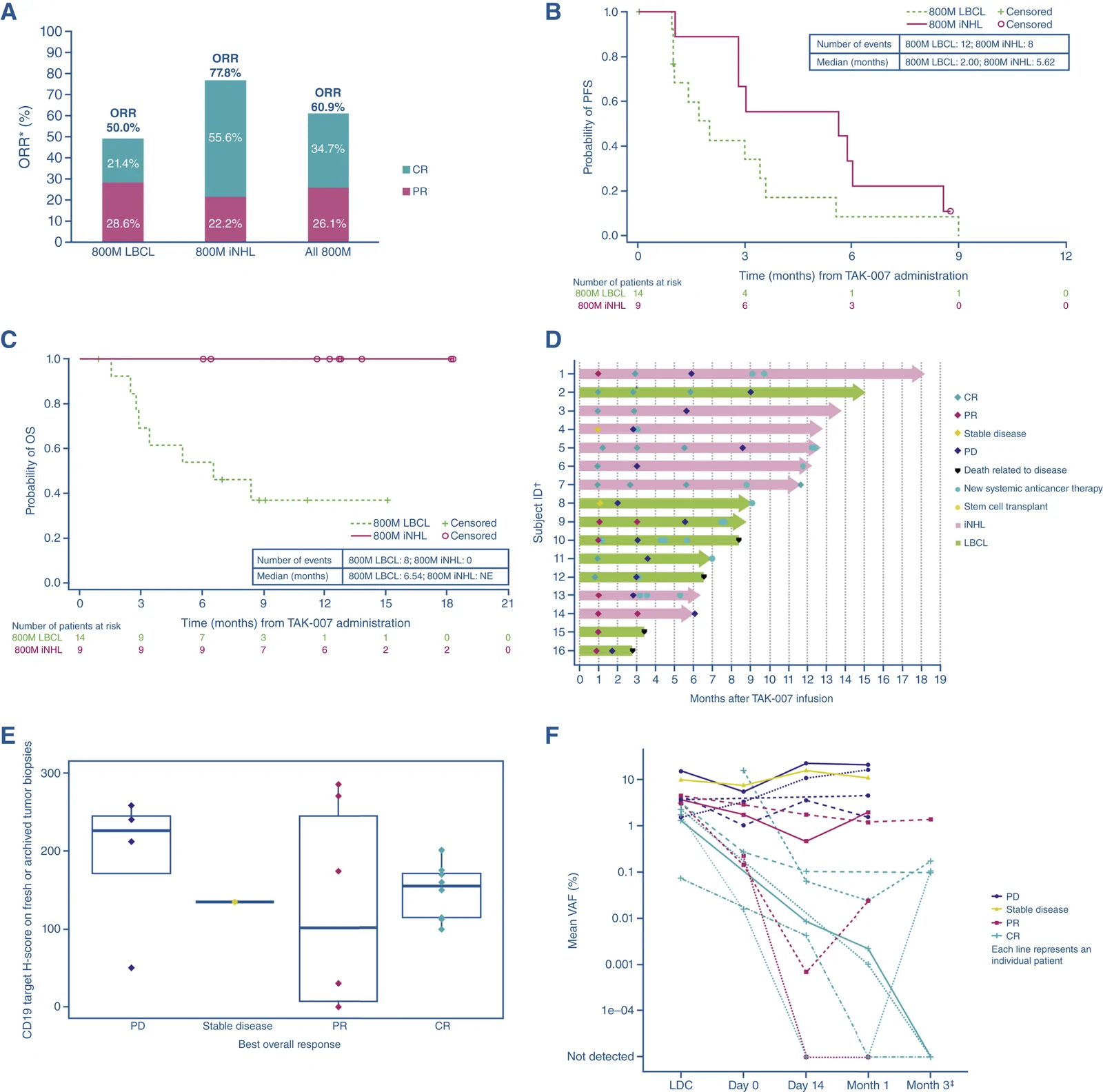
Efficacy analysis conducted in 800M escalation and expansion cohorts. **A,** Clinical responses at 1 month. **B,** Kaplan–Meier plot of PFS in 800M dose escalation and expansion cohorts. **C,** Kaplan–Meier plot of OS in 800M dose escalation and expansion cohorts. **D,** DOR in 800M dose escalation and expansion cohorts. **E,** Clinical response across varying target antigen levels measured by immunohistochemistry on tumor biopsies. **F,** Mean VAF % over time. Each line represents an individual patient. *ORR is defined as the incidence of CR and PR. ^†^Includes patients who had a response at month 1. ^‡^One patient had a longitudinal sample with a ctDNA result at month 2, plotted here along with the data points labeled as month 3. PD, progressive disease.

In the 800M escalation and expansion cohorts, the ORR by investigator assessment was 60.9% [95% confidence interval (CI), 38.5 to 80.3], with complete response (CR) and partial response (PR) achieved by eight (34.7%) and six (26.1%) patients, respectively (Fig. 2A). The ORR was 50% (95% CI, 23 to 77) and 77.8% (95% CI, 40 to 97.2) in the 800M LBCL and iNHL cohorts, respectively. In the 800M LBCL cohort, 3 (21.4%) patients had CR and 4 (28.6%) had PR. In the 800M iNHL cohort, CR was achieved by 5 (55.6%) patients, and 2 (22.2%) patients had PR. Among seven patients who previously received CD19 CAR T cells and TAK-007 800M, three (43%) had a response including two (29%) patients with CR. No responses were reported in the patients who received TAK-007 200M.

Median PFS by an investigator assessment was 2 months (95% CI, 0.99 to 3.58) in the 800M LBCL cohort and 5.6 months (95% CI, 1.05 to 8.57) in the 800M iNHL cohort (Fig. 2B). As of data cutoff, 10 (38.5%) patients had died: two, three, and five patients in the 200M escalation cohort, 800M escalation cohort, and LBCL 800M dose expansion cohort, respectively. The median overall survival (OS) was 6.5 months [95% CI, 2.79 to not estimated (NE)] in the 800M LBCL cohort and NE in the 800M iNHL cohort (Fig. 2C). The median duration of response (DOR) was 2.6 months (95% CI, 0.85 to NE) in the 800M LBCL cohort and 4.9 months (95% CI, 1.84 to 7.39 months) in the 800M iNHL cohort. Figure 2D shows the DOR of patients in the 800M LBCL and iNHL dose escalation and expansion cohorts. Clinical responses were observed across varying target antigen levels, demonstrating that responses to TAK-007 were independent of CD19 expression status (Fig. 2E). Two patients with a CD19 H-score of 0 were observed to have a PR, which could be attributable to innate NK receptor–mediated cytotoxicity, given the lack of detectable CAR target antigen. Metabolic tumor volume (MTV) at screening by response is shown in Supplementary Fig. S1A–S1C. The mean MTV was 279.2 cm^3^ in the 800M LBCL cohort (Supplementary Fig. S1B) and 548.5 cm^3^ in the 800M iNHL cohort (Supplementary Fig. S1C).

In total, 19 patients received subsequent anticancer therapies: *n* = 2 in the 200M escalation cohort, *n* = 3 in the 800M escalation cohort, *n* = 9 in the 800M LBCL expansion cohort, and *n* = 5 in the 800M iNHL dose expansion cohort.

### Circulating Tumor DNA Analysis

The analysis revealed that baseline circulating tumor DNA (ctDNA) levels [% variant allele frequency (VAF)] were higher in nonresponders compared with responders, suggesting a greater tumor burden among nonresponders. Following LDC, a reduction in ctDNA was observed in the majority of patients.

Responders showed a decline in ctDNA levels from day 0 to day 14 and month 1 following treatment compared with nonresponders, underscoring the effectiveness of TAK-007 in reducing ctDNA tumor burden, as illustrated in Fig. 2F. All patients with CR (6 of 6 tested) and PR (4 of 4 tested) exhibited a reduction in % VAF in their first available longitudinal sample post-treatment, as early as 2 weeks after TAK-007 treatment, in comparison with baseline levels. However, a subset of responders (3 of 10) showed an increase in ctDNA levels between months 1 and 3, possibly indicating disease relapse. All three patients that had undetectable ctDNA levels at month 3 had a best response of CR and DOR of >4.5 months; two patients had disease progression at or after 6 months, and one patient received a subsequent anticancer therapy. Overall, a ctDNA-negative response was observed in five patients (four with CR and one with PR), demonstrating that a deep molecular response is achievable with an allogeneic CAR-NK product.

### CK and Pharmacodynamics

**Figure 3.**
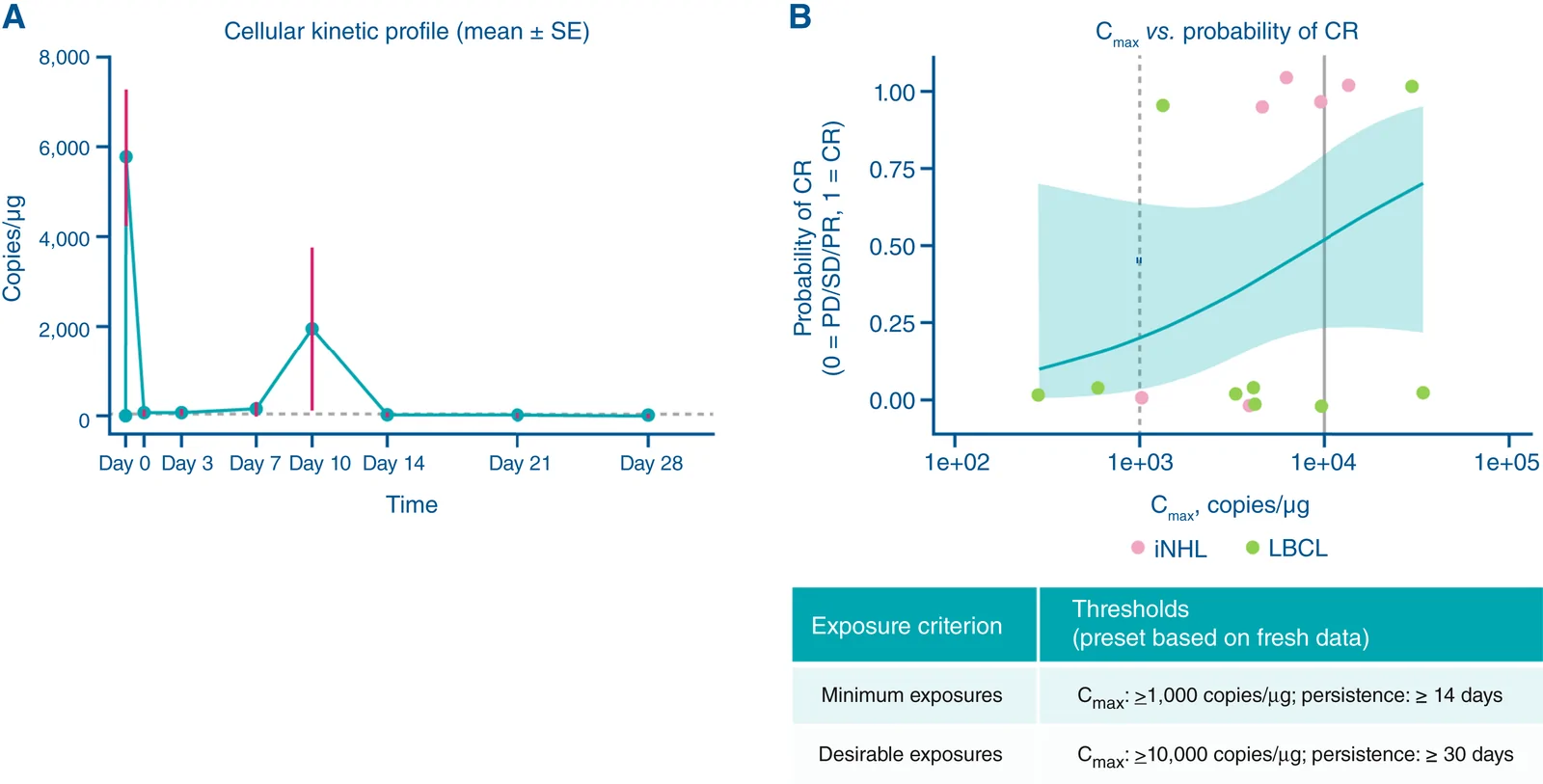
Cellular kinetics conducted in the pharmacodynamic set. **A,** CK profile [mean ± standard error (SE)] in patients receiving TAK-007 (800M). **B,** Logistic regression of the probability of complete response. The blue line indicates the predicted response (*P* value using the Wald test: 0.45), whereas the light blue ribbon indicates the 95% CI of the predicted response. Individual C_max_ values are shown as colored circles. The minimum exposure thresholds were defined as peak levels (C_max_) of ≥1,000 copies/μg after infusion, with persistence as quantifiable levels for a minimum of 14 days. The desirable exposures associated with meaningful efficacy threshold were defined as peak levels (C_max_) of ≥10,000 copies/μg and persistence for at least 30 days. PD, progressive disease; SD, stable disease.

TAK-007 CK exhibited a multiphasic disposition, characterized by an initial cellular contraction, followed by a cellular expansion (Fig. 3A). Peak serum levels were observed on days 7 to 10 following TAK-007 administration, which were followed by a terminal elimination phase with decreasing levels after day 10. The geometric mean maximum observed blood plasma concentration (C_max_) and area under the concentration-time curve from time 0 to the time of the last quantifiable concentration (AUC_last_) of TAK-007 were 5,010 copies/μL (111%) and 549 days × copies/μL (10,100%) in the 800M LBCL dose expansion cohort and 3,460 (710%) copies/μL and 4,900 days × copies/μL (100,000%) in the 800M iNHL dose expansion cohort, respectively. There was a trend suggesting that a higher C_max_ of TAK-007 may be associated with an increased response rate in patients in the 800M LBCL and iNHL dose expansion cohorts (Fig. 3B).

No significant elevation in plasma IL6 levels was observed following TAK-007 administration, which is consistent with the absence of grade ≥3 CRS and ICANS. There were no significant increases in the probability of AEs with increased C_max_ of TAK-007 (Supplementary Fig. S2).

The majority of patients were B-cell aplastic (<50 B cells/μL of blood) at baseline and showed persistent B-cell depletion through 3 months after treatment (Supplementary Fig. S3). Replication competent retrovirus (RCR) positivity was not detected in any of the analyzed patient samples (*n* = 26). Supplementary Figure S4A to S4H shows the profiles of interferon γ, IL12, IL10, IL8, granulocyte–macrophage colony-stimulating factor, and tumor necrosis factor (TNF) following TAK-007 administration. Increases in plasma IL15 were observed after LDC in all cohorts. IL15 levels remained elevated 1 day after TAK-007 administration and decreased by day 7, continuing to decline thereafter with a return to baseline levels by month 2 in all cohorts (Supplementary Fig. S4A).

### Immunogenicity

No patients had detectable anti-CAR antibodies present at the time of TAK-007 administration, and no emergence of anti-CAR antibodies was observed after treatment. Anti-HLA antibodies specific to the donor were only detected in three patients: one in the 800M escalation cohort and two in the 800M iNHL dose expansion cohort. For the patient in the 800M escalation cohort, a baseline sample was unavailable, and donor-specific anti-HLA antibodies were only detected at month 12. In the 800M iNHL expansion cohort, one patient had pre-existing anti-HLA antibodies. The other patient had donor-specific anti-HLA antibodies that were detected on day 14 and month 2 (Supplementary Table S2). A baseline result was unavailable for this patient, confounding the interpretation of whether this event was treatment emergent. Overall, no significant treatment-emergent anti-CAR or donor-specific anti-HLA antibodies were detected.

### Product Characteristics and Clinical Responses

All product lots used in this study were manufactured from CB units (CBUs) that met the criteria for optimal CBUs as outlined in Marin and colleagues (14). In order to identify additional factors potentially correlating to clinical responses observed, we performed deep molecular characterization of the drug product at various stages of manufacturing using single-cell RNA sequencing (scRNA-seq). For the correlative analysis, the six individual drug product lots used for dosing the study patients were classified as responsive (eliciting a clinical response) versus nonresponsive (not eliciting a clinical response) following TAK-007 treatment as described in Supplementary Table S3.

**Figure 4.**
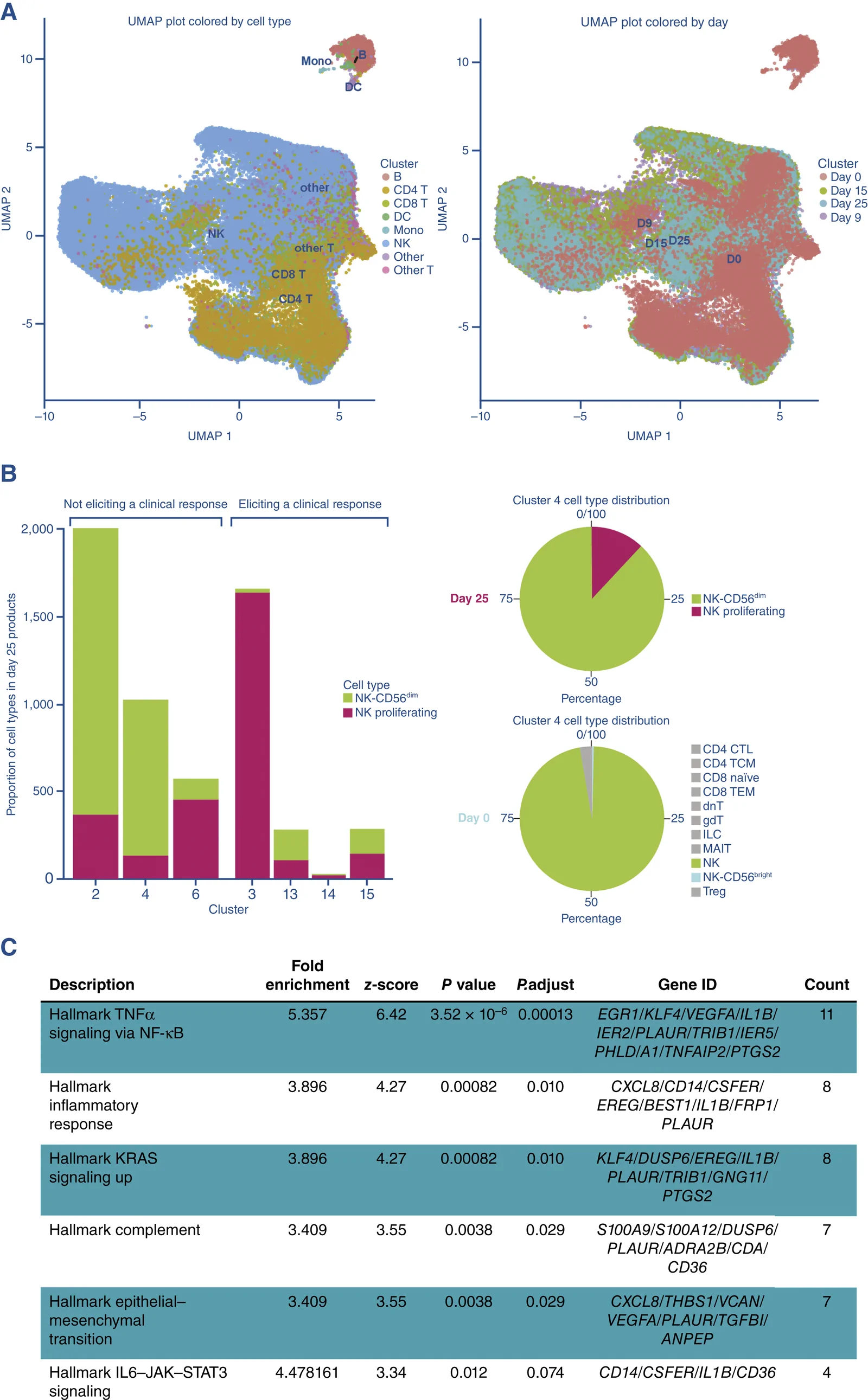
Product characteristics and clinical responses. **A,** Clusters identified by scRNA-seq (53 samples in total were assayed on days 9, 15, and 25 during TAK-007 manufacturing). Eight donors were sampled at baseline (day 0) and subsequently split into CAR and non-CAR culture conditions, with measurements taken at three timepoints (days 9, 15, and 25). This corresponds to 8 baseline samples and 16 samples at each subsequent timepoint under ideal conditions. However, on day 9, three donors did not have paired samples for both conditions, resulting in a total of 8 baseline samples and 13, 16, and 16 samples at days 9, 15, and 25, respectively. The cell types were defined using the Azimuth database (https://azimuth.hubmapconsortium.org/references/#Human%20-%20PBMC). “Other” and “other T” are the fallback/low confidence categories indicating that cells did not map to a known cell type with confidence. **B,** Clusters identified by scRNA-seq associated with clinical responses. Six drug product lots were analyzed, including two that did not elicit a clinical response and four that did. A higher proportion of NK proliferating cells in product lots elicited a response as shown in clusters 3, 13, 14, and 15. Clusters 2, 4, and 6 show those product lots not eliciting a clinical response. Product lot details are provided in Supplementary Table S3. **C,** Pathways associated with nonresponsive samples (at day 0): overrepresentation analysis showing that genes in hallmark pathways TNFα, inflammatory response, and KRAS signaling are enriched. CTL, cytotoxic T lymphocytes; dnT, double-negative T-cells; gdT, gamma-delta T-cells; ILC, innate lymphoid cells; MAIT, mucosal-associated invariant T-cells; TCM, central memory T-cells; TEM, effector memory T-cells; Treg, regulatory T cells.

Clustering analysis revealed distinct separation of the single cell clusters by the stage of manufacturing, with day 0 samples predominantly consisting of T and B cells and day 25 samples of NK cell subsets, as expected, with gradual transitions at day 9 and day 15 of the manufacturing process. Similar results were observed in untransduced samples. Detailed cluster and cell type annotations are presented in Fig. 4A.

Interestingly, the four drug product lots that elicited a clinical response were observed to have lower levels of exhausted NK-CD56^dim^ cells and higher levels of NK proliferating cells annotated using Azimuth (RRID: SCR_021084), a public database of curated human peripheral blood mononuclear cell types (Fig. 4B; refs. 16, 17). The Azimuth database characterizes cells with the NK-CD56^dim^ label as CD16-positive, CD56^dim^, and expressing markers *GNLY*, *TYROBP*, *NKG7*, *FCER1G*, *GZMB*, *TRDC*, *PRF1*, *FGFBP2*, *SPON2*, and *KLRF1*. In contrast, NK proliferating cells are characterized as typically expressing *MKI67*, *KLRF1*, *TYMS*, *TRDC*, *TOP2A*, *FCER1G*, *PCLAF*, *CD247*, *CLSPN*, and *ASPM*. We further investigated cell clusters differentially enriched in CAR-NK day 25 product lots, distinguishing those eliciting responses from those that did not. Clusters 2, 4, and 6 were enriched in lots that did not elicit a clinical response, with clusters 2 and 4 mainly comprising NK-CD56^dim^ cells traceable to day 0, in which cluster 4 had the greatest abundance (Fig. 4B). This suggests that a higher proportion of NK proliferating cells might serve as a criterion for selecting donor material predictive of patient response.

To understand whether fetal determinants influence responsiveness (as described earlier by Marin and colleagues; ref. 14), we performed a pseudobulk analysis of scRNA-seq at day 0, comparing gene expression in product lots that elicited a clinical response and in those that did not. The top 25 differentially expressed genes in day 0 samples are represented in a heatmap (Supplementary Fig. S5). Genes associated with products that do not elicit a clinical response are enriched in TNFα-mediated NF-κB expression, inflammatory response, and *KRAS* upregulation (Fig. 4C). The most significantly enriched pathway in genes upregulated in products that did not elicit a clinical response was observed to be TNFα-mediated NF-κB expression (*EGR1*, *KLF4*, *VEGFA*, *IL1B*, *IER2*, *PLAUR*, *TRIB1*, *IER5*, *PHLDA1*, *TNFAIP2*, and *PTGS2*; Fig. 4C).

## Discussion

In this open-label, multicenter, phase II study, which investigated the safety and efficacy of TAK-007 in heavily pretreated adult patients with r/r B-cell NHL, TAK-007 800M was identified as a suitable RP2D after no DLTs were observed during dose escalation. The overall safety profile of TAK-007 was consistent with the anticipated safety profile of a CAR NK-cell therapy (14, 15). Similar to the results from a phase I/II study that utilized fresh anti-CD19 CAR-NK cells manufactured with the same genetic construct and NK source as TAK-007 but cultured for only 14 days, most TEAEs observed in the present study were hematologic in nature and attributable to LDC (14, 15). Neurological toxicities, ICANS, CRS, and GvHD are significant clinical concerns for autologous and allogeneic CAR T therapies (1–4, 8, 9). Importantly, we observed no events of GvHD, ICANS, or neurotoxicity, supporting the tolerable safety profile of TAK-007. Although there were no cases in the previous phase I/II study, three patients in the present study experienced TAK-007–related grade 1 to 2 CRS. However, the observed CRS events may also have been related to potential infections in these patients. It is notable that the main toxicity observed was driven by LDC, with a higher proportion of patients who experienced grade ≥3 TEAEs related to lymphodepletion only (84.6%) compared with TAK-007 only (16.7%), highlighting the tolerable nature of TAK-007.

The ORR of 60.9% observed across the TAK-007 800M dose escalation and expansion cohorts was similar to the ORR of 49% from the aforementioned phase 1/2 study with fresh CD19 CAR-NK cells (14, 15). However, a more relevant comparison is with the subset of patients in that trial who received CAR-NK cells manufactured from “optimal” CB units, defined as units cryopreserved within 24 hours and with low nucleated red blood cell content. This subgroup demonstrated a notably higher 1-year PFS rate of 68% (14, 15). Although TAK-007 was also derived from similarly selected cord units, its lower observed response rate may reflect the effects of prolonged 25-day *ex vivo* expansion and cryopreservation. Although these manufacturing modifications enable off-the-shelf availability and scalability, they could potentially influence NK-cell potency or persistence *in vivo*.

With TAK-007, the ORR, CR, DOR, and PFS were numerically higher in the 800M iNHL escalation and expansion cohort compared with any other cohort, similar to the results of the phase I/II study, indicating that patients with less aggressive disease may be more likely to respond to treatment (14). In the TAK-007 800M dose expansion and escalation cohorts, 8 (34.8%) patients achieved CR. Of those, 3 (13%) and 5 (21.7%) were in the 800M LBCL and iNHL cohorts, respectively. Importantly, TAK-007 was shown to drive clinical responses across varying target antigen levels, relevant to a real-world setting. No responses were observed at the lower 200M dose level, indicating that efficacy may be dose-dependent. Of note, patients in the current study were heavily pretreated with a median of 5 and up to 11 prior lines of therapy. In addition, 42.3% of patients had previously received CD19-targeting therapies including 42.9% of the 800M LBCL cohort pretreated with CD19-targeting autologous CAR T cells. Taken together, this demonstrates the effectiveness of TAK-007, even as a potential later-line therapy.

The exposure-response analysis indicated a positive correlation between higher exposures of TAK-007 (C_max_) and response to treatment (CR and ORR) which is supported by previous studies (14, 15). Our analysis demonstrated that ctDNA-negative responses are achievable with a single infusion of TAK-007, which are typically observed with CAR T-cell treatment for B-cell NHLs (18). Reductions in ctDNA levels were observed as early as 2 weeks following TAK-007 treatment in clinical responders. Several studies in LBCL demonstrated similar findings of lower ctDNA responses after treatment being associated with higher CR and PR rates and longer PFS (18–20). A large decrease of ctDNA was reported in patients with LBCL who achieved a complete or partial metabolic response 4 to 6 weeks post-CAR T therapy, further supporting our findings (20). Dynamic monitoring of ctDNA is therefore a potential noninvasive method to predict treatment response and survival outcomes for cell therapies (21), with the ability to facilitate the development of adaptive redosing strategies. Additionally, baseline ctDNA levels were significantly higher in nonresponders compared with responders, indicating that patients with a higher tumor burden may have a lower likelihood of response. This limitation may be overcome with higher dosing of TAK-007 or prior tumor debulking.

Persistence of CAR-NK cells was notably shorter with TAK-007 than in the earlier trial with fresh CAR-NK cells (14, 15). In that study, infused cells were detected in patients for up to a year (14, 15), whereas in the present study, peak serum levels were observed on days 7 to 10 after TAK-007 administration, followed by a terminal elimination phase with decreasing levels after day 10. This shorter persistence may be attributed to extended culture duration and cryopreservation, both of which are known to influence NK cell viability and metabolic fitness (22). Moreover, these factors, in addition to the extensive prior treatments received by patients, might have contributed to the reduced durability of clinical responses observed in this study. Thus, there is a broader need to explore optimization of CAR-NK cell manufacturing and dosing regimens to address the challenge of short DORs and to achieve sustained longer-term remissions. Strategies to mitigate exhaustion during culture, such as shorter duration of culture, improved cryopreservation techniques, and enhanced post-thaw functionality, are critical to achieving sustained remissions.

The majority of patients in this study (73.1%) were able to receive subsequent anticancer therapies following TAK-007 administration, highlighting its favorable safety profile. In addition, there were no significant treatment-emergent anti-HLA or anti-CAR antibodies, and no RCR positivity was detected. Taken together, the manageable immunogenicity and safety profiles may suggest that there is potential for multiple doses without a significant risk of antidrug antibodies that could negatively impact subsequent doses. As a future direction, the potential for multiple administrations of TAK-007, as well as its use in combination with agents such as cytokines, warrants further investigation, given the favorable safety profile of TAK-007 and prior reports supporting the feasibility of these approaches (23–25).

TAK-007 utilizes cryopreserved cells from healthy donors instead of patients, which are manufactured before an individual requires therapy. Providing treatment in a timely manner could reduce long turnaround times of traditional CAR T-cell therapies and negate the need for bridging therapies (6, 15). TAK-007 was available within a median of 7 days at the study site after submission of the order request, highlighting efficient and quick delivery as well as off-the-shelf availability. Additionally, the use of cryopreserved cells can overcome the limitations related to existing cytopenia and T-cell dysfunction (6). Furthermore, the lack of major safety concerns with TAK-007 may allow administration in outpatient settings, thereby reducing financial and geographical burdens on patients (15).

In the previous study on CAR-NK therapy by Marin and colleagues (14), which involved a shorter duration of manufacturing (14 days) and infusion of fresh CAR-NK cells as described above, it was observed that clinical outcomes were significantly improved when CAR-NK cells were derived from a CBU with nucleated red blood cells ≤8 × 10^7^ and a collection-to-cryopreservation time of ≤24 hours. NK cells from these optimal CBUs demonstrated enhanced functionality and increased expression of effector-related genes. In our TAK-007 CAR-NK study, all the drug product lots used for clinical patient dosing were derived from such optimal CBUs. The manufacturing modifications of TAK-007 enable batch production of multiple doses, providing scalability and logistical flexibility; however, the extended 25-day manufacturing process and subsequent cryopreservation may have mitigated some of these advantages.

Deep molecular characterization of TAK-007 revealed enrichment of NK proliferating cells in products eliciting clinical response, whereas NK-CD56^dim^ cell clusters were found in products that did not. Our analysis further supports that enrichment of specific NK-cell subsets in the starting material or drug product can enhance clinical responses and serve as a criterion for selecting donor material predictive of patient response, as well as guide product selection. Because products derived from a single CB donor could be used to dose multiple patients, selection of NK cell subsets with high proliferative potential during the manufacturing process could enable development of a more potent, “enriched” final product. Larger prospective studies are needed to determine whether this approach may allow for the administration of fewer but more effective NK cells, potentially enhancing patient responses. The most significantly enriched pathway in genes upregulated in products that did not elicit a clinical response was TNFα-mediated NF-κB expression. This suggests a transcriptional program associated with activation-induced stress and inflammation likely compromising NK-cell function (14). Additional studies with a larger sample size are needed to validate these findings and gain deeper insight into the role of these genes in TAK-007 function. Similarly, Marin and colleagues (14) reported that Sub-C NK cells associated with worse outcomes showed activation of pathways related to inflammation, hypoxia, apoptosis, TNFα signaling via NF-κB response, and DNA damage.

The protocol initially consisted of two parts: part 1 (dose escalation and expansion) and part 2 (efficacy). A data-driven decision was made to close enrollment after part 1, and part 2 was subsequently removed from the protocol. Although the findings of this study were limited by the small number of patients, this study served to better characterize the safety profile of a single dose of TAK-007, allowing us to assess potential risks known to be prevalent with similar therapies and, in addition, identify the RP2D. This study also demonstrated that TAK-007 treatment offers potential clinical and operational advantages over traditional CAR T-cell therapies. Although TAK-007 administered at 800M in the LBCL and iNHL populations showed preliminary efficacy, further studies are needed to optimize outcomes by refining manufacturing protocols, including shortening the duration of cell expansion, an approach being actively pursued in CAR T-cell therapies, and improving cryopreservation techniques to enhance cell functionality and persistence. Additionally, exploring reinfusion schedules and combination strategies could further improve therapeutic efficacy.

## Methods

### TAK-007 Manufacturing and Cryopreservation

The manufacturing process for TAK-007, adapted from the original method developed at MD Anderson Cancer Center (MDACC; refs. 14, 15), incorporates key modifications to improve consistency and scalability. Notably, the culture duration was extended from 14 to 25 days to increase cell yield and enable manufacturing scale-up cryopreservation to produce an “off-the-shelf” allogeneic CAR NK-cell therapy product. Umbilical CBUs are selected in compliance with United States Food and Drug Administration regulations and based on specific criteria described in Marin and colleagues (14), which include time from collection to cryopreservation of ≤24 hours and pre-freezing nucleated red blood cells of ≤8 × 10^7^ cells. All CBUs used for the manufacturing of TAK-007 fulfilled these criteria.

The CBUs for TAK-007 manufacturing were obtained from the MD Anderson Cord Blood Bank. CBUs were thawed, and NK cells were enriched using automated magnetic selection. The enriched NK cells were then activated with universal antigen-presenting cells (uAPC) and IL2. The enriched NK cells were transduced with a γ-retroviral vector encoding anti-CD19 CAR, IL15, and inducible caspase 9 (14, 15). The uAPCs, derived from the K562 cell line, were used as feeder cells to enhance NK-cell proliferation and persistence during co-culture, as previously described (12). The uAPCs were irradiated at 100 Gy to prevent replication, cryopreserved, and thawed immediately prior to use. These genetically modified cells were utilized exclusively for *ex vivo* expansion of CB-derived NK cells and were not intended for patient infusion.

Post-transduction, the CAR-NK cells were expanded until day 25 and then cryopreserved. The expansion duration used was longer compared with the harvesting of CAR-NK cells on day 14 in the original MDACC protocol to allow higher cell yields. Preclinically, no differences were observed between CAR-NK cells expanded for 14 and 25 days. The process is semiclosed and semiautomated, minimizing contamination risks and ensuring product consistency. In-process controls and quality checks were implemented throughout to monitor cell viability, purity, and identity. Additional details about *in vitro* and *in vivo* characterization of cryopreserved and fresh products are provided in the Supplementary Materials.

Cryopreservation of CAR-NK cells was performed at a defined target concentration in cryobags, and the cells were suspended in a cryopreservation medium containing a pH-controlled buffer, protein stabilizer, and permeating cryoprotectant. The CAR-NK cells were frozen using a controlled-rate freezer with their optimal freezing program and then stored in liquid nitrogen vapor phase. Cryopreserved materials were shipped under cryogenic conditions using liquid nitrogen dry shippers to maintain temperatures during transport.

### *In Vitro* and *In Vivo* Characterization of Cryopreserved and Fresh Product

The previous phase I study performed at MDACC used fresh product harvested either at day 15 (with two rounds of uAPC stimulations) or day 22 (with three rounds of uAPC restimulation).

Development of the cryopreserved TAK-007 product began with an evaluation of the impact of NK-cell activation status on post-thaw product potency. NK cells were harvested at different timepoints following uAPC stimulation, and post-thaw *in vitro* cytotoxicity was assessed (Supplementary Fig. S6A). Cryopreserved products harvested 9 to 10 days after uAPC stimulation (days 18 or 25) demonstrated better preservation of potency compared with earlier harvests (days 15 or 22), independent of the number of uAPC stimulation rounds.

A direct comparison of day 18 and day 25 harvests was subsequently performed using both the manual process (similar to the phase 1 manufacturing process) and the newly developed automated process for TAK-007 (Supplementary Fig. S6B). As no significant differences were observed between the two harvest times, day 25 was selected as the standard harvest point to increase batch yield.

Additional optimization methods, including the evaluation of cryoformulation components, large-volume (50 mL) cryovial fill-finish procedures, controlled-rate freezing, and thawing conditions, were conducted to establish the final cryopreservation process for TAK-007. CD19 CAR-NK cells for both fresh control and cryoproduct were co-incubated with Raji tumor cells at seven different effector-to-target cell ratios (CAR-NK cells or TAK-007 to Raji cells; E/T ratios 10:1, 5:1, 2.5:1, 1.25:1, 0.625:1, 0.312:1, and 0.156:1). The Raji cells were prelabeled with a fluorescent dye (CellTrace Violet) to allow their discrimination from the effector cells. After a 4-hour incubation period, the killing activity of the different effector cells at different E/T ratios was determined by flow cytometry, with dead target cells identifiable by positive caspase 3/7 expression and SYTOX AADvanced dead cell staining. *In vivo* testing in the Raji B-cell lymphoma model showed that the cryopreserved product retained efficacy comparable with fresh material (Supplementary Fig. S6C).

A similarity analysis was conducted comparing cryopreserved TAK-007 [*N* = 6 lines of therapy (LoT)] with fresh phase 1 CD19 CAR-NK (*N* = 26 LoTs) based on post-thaw viability, NK-cell purity, CAR percentage, vector copy number (VCN), and *in vitro* cytotoxicity (Supplementary Fig. S6D). The post-thaw viability of TAK-007 was measured using NC-200 following the vendor’s instructions. The CAR% and NK cell purity were assessed using the flow cytometry method previously described. The VCN of TAK-007 was generated using qPCR and the droplet digital polymerase chain reaction (ddPCR) method with specific primers against TAK-007 and a reference primer. The fresh CD19 CAR-NK product analysis methods were elaborated in the previous publication (14). No significant differences were observed. These studies may have been limited in their ability to detect differences due to assay setup.

### Trial Design and Patients

This was an open-label, multicenter, phase II study of TAK-007 in patients with r/r B-cell NHL, including LBCL and iNHL who had failed ≥2 prior systemic therapies which may have included prior autologous CAR T-cell therapy (NCT05020015; Fig. 1C). Patients were enrolled across nine sites in the US. During the dose escalation phase, patients received TAK-007 at two dose levels: 200 × 10^6^ or 800 × 10^6^ CD19 CAR+ viable NK cells, hereby referred to as 200M or 800M, respectively.

A sequential dose escalation guided by Bayesian Optimal Interval (BOIN) design was used, followed by expansion cohorts to determine the RP2D (26–28). The BOIN design used the following rule, optimized to minimize the probability of an incorrect dose being assigned and to guide dose escalation/de-escalation:

- If the observed DLT rate at the current dose was ≤0.197, the dose should be escalated to the next higher dose level; if the current dose was the highest dose, the new patients should be treated at the highest dose.
- If the observed DLT rate at the current dose is >0.298, the dose should be de-escalated to the next lower dose level; if the current dose is the lowest dose and the rule indicates dose de-escalation, the new patients should be treated at the lowest dose unless the number of DLTs reaches the elimination boundary at which point the dose escalation should be terminated for safety.
- Otherwise, stay at the current dose.
- The above should be repeated until the maximum sample size of 12 is reached, or until the number of evaluable patients treated at the current dose reaches 6; the decision according to the above was to stay at the current dose.

The dose expansion phase comprised separate expansion cohorts for LBCL and iNHL, with patients receiving TAK-007 800M. The dose escalation and expansion cohorts were determined using previously reported criteria (14). Treatment included three consecutive days of intravenous LDC with cyclophosphamide 300 mg/m^2^ of body surface area (BSA) and fludarabine 30 mg/m^2^ of BSA, followed by a single dose of TAK-007 administered at least 2 days later. The TAK-007 product was provided as a cryopreserved suspension of living cells that was thawed before administration.

Eligible patients were aged 18 years and older, diagnosed with previously treated r/r histologically proven CD19-expressing LBCL or iNHL, with a life expectancy of ≥12 weeks, who had measurable disease defined as at least one lesion per the Lugano classification and an Eastern Cooperative Oncology Group performance score of 0 or 1. Patients were required to have previously received at least two prior lines of therapy, including an anti-CD20 monoclonal antibody and chemotherapy regimen. Patients who received CD19-targeted therapy including CD19 CAR T cells were permitted to be enrolled 3 months after the last therapy. A full listing of inclusion and exclusion criteria is available on ClinicalTrials.gov (identifier: NCT0502001; ref. 29).

The study was conducted in accordance with the International Council for Harmonization Good Clinical Practice standards, the Declaration of Helsinki, and all applicable regulatory requirements. Relevant institutional review boards or ethics committees approved all aspects of the study, and all authors had access to primary clinical trial data. All patients provided written informed consent.

### Objectives and Endpoints

The primary objectives were to determine the safety and tolerability and establish the RP2D of TAK-007 in adults with r/r B-cell NHL. The primary endpoints for this study were the incidence of AEs and clinically significant laboratory values and vital signs. The secondary objectives were to evaluate efficacy endpoints including ORR, CR, DOR, PFS, and OS per investigator assessment. Secondary objectives also included characterization of TAK-007 CK parameters, including C_max_ and AUC_last_, pharmacodynamic biomarker assessments using B-cell quantification and cytokine profiling, RCR testing, and assessment of the immunogenicity of TAK-007 through prevalence and incidence of antidrug antibodies (anti-HLA and anti-CAR). Exploratory objectives included population CK and pharmacodynamic modeling to further evaluate the relationship between exposure and response, to investigate biomarkers of clinical response and assessment of ctDNA, and to determine the cellular product characteristics in drug product batches based on whether they elicited a clinical response or not.

### Study Assessments

Occurrence of DLTs was assessed during the dose escalation phase in patients who received TAK-007. DLTs were defined as grade ≥3 non-hematologic TEAEs clearly unrelated to the underlying disease and at least possibly related to the investigational therapy (TAK-007 and/or lymphodepletion) during the dose escalation phase and within 28 days of TAK-007 administration with the following exceptions:

- Asymptomatic laboratory changes (other than renal and hepatic laboratory values) that could be successfully managed (reversal of grade 4 events to grade ≤2; reversal of grade 3 events to grade ≤1 or baseline) within 72 hours.
  - Grade 3 nausea/vomiting that could be managed subsequently with antiemetics (grade 3 nausea or vomiting that persisted beyond 72 hours with or without appropriate medical intervention was considered a DLT).
  - Grade 3 fatigue lasting <7 days.
  - Grade 3 diarrhea lasting <72 hours.
  - Grade 3 renal and hepatic toxicity that improved to grade ≤2 within 7 days.
  - Grade 3 infusion-related reactions that responded to symptomatic treatment without recurrence of grade 3 symptoms.
  - Grade 3 tumor lysis syndrome (TLS) lasting ≤14 days and grade 4 TLS lasting ≤7 days.

Grade ≥3 hematologic TEAEs that were clearly not attributable to previous lymphoma therapy and/or marrow infiltration related to the underlying disease and at least possibly related to the investigational therapy (TAK-007 and/or lymphodepletion) were considered DLTs with the following exceptions:

- Grade ≥3 neutropenia lasting <21 consecutive days.
- Grade 3 febrile neutropenia lasting <14 days.
- Grade ≥3 thrombocytopenia lasting <21 consecutive days.
- Cytopenia except neutropenia and thrombocytopenia as described above.

Grade 2 acute GvHD requiring systemic steroids and not resolving within 7 days.

Safety was assessed by continuous monitoring of AEs and regularly scheduled assessments for vital signs and clinical laboratory evaluations. TEAEs were graded according to the National Cancer Institute Common Terminology Criteria for Adverse Events Version 5.0 (30) except for CRS and ICANS which were graded according to American Society for Transplantation and Cellular Therapy guidelines (31) and GvHD which was graded according to consensus guidelines (32, 33).

Disease assessments were conducted by radiographic evaluation consisting of ^18^F-fluorodeoxyglucose-positron emission tomography imaging and/or contrast-enhanced computed tomography. The response was assessed locally according to the International Working Group 2014 Lugano classification (34). A bone marrow biopsy to confirm CR was performed for patients with iNHL at the time of radiographic CR if they had initial bone marrow involvement.

### ctDNA Analysis

Genomic profiling and ctDNA analysis were performed using the Foresight Diagnostics MRD platform PhasED-seq technology as previously described in Klimova and colleagues (35). Plasma samples were collected at lymphodepletion, day 0 (infusion), day 14, month 1, and month 3 post TAK-007 infusion. Changes in levels of ctDNA measured as a percent variant allele frequency were assessed over time.

### Immunogenicity

Anti-TAK-007 CAR extracellular domain (FMC63) antibodies were detected using a custom electrochemiluminescence bridging assay in patient serum samples. The method used acid dissociation of ADA-free drug complexes to allow for preferential capture of ADA in the presence of drugs. Biotinylated, ruthenylated-drug conjugates and ADA complexes were captured and detected using a Meso Scale Discovery (MSD) reader. The presence of ADA was assessed by a 3-tier approach of screening, confirmatory, and tier-assay formats. Anti-HLA antibodies were detected using flow cytometry and Luminex platforms for the presence of antibodies against HLA class I and II antigens in serum, and donor-specific antibodies were defined using the HLA typing results from a next-generation sequencing assay.

### CK and Pharmacodynamic Biomarkers

CK was measured using ddPCR by relative quantification of the TAK-007 transgene in whole blood. TAK-007 CAR-NK copy numbers are reported as copies/μg using the reference gene copies/reaction. DNA isolated from whole blood collected from patients was utilized for this analysis.

B-cell counts were measured by flow cytometry in whole blood. Circulating cytokines were measured using MSD in patient plasma samples collected at specified timepoints per study protocol.

### RCR Testing

RCR was detected using a custom multiplex ddPCR assay developed for quantitation of RCR relative to a reference gene. DNA isolated from whole blood collected in K2EDTA from patients was utilized for this analysis.

### Product Characterization

scRNA-seq was performed on the final product (day 25) and intermediates from various stages of manufacturing (days 0, 9, and 15). After quality control (QC), the scRNA-seq data were normalized using the standard normalization and scaling functions in Seurat version 5.1.0. The Seurat anchor gene-based canonical correlation analysis was used to integrate multiple datasets and correct for batch effects. Annotation of cell types was based on the Azimuth database for peripheral blood mononuclear cell subsets (2). Annotation was completed to level 2, which contained three NK cell subtypes (NK-CD56^dim^, NK proliferating, and NK-CD56^bright^). The Fisher exact test was applied to determine enrichment of cell clusters in product material that did and did not elicit a response. The scRNA-seq read counts are available at https://zenodo.org/records/19615830.

### scRNA-seq QC

Initial quality control of the scRNA-seq data was performed to filter out low-quality cells. The following criteria were applied:

- Minimum genes per cell: 200.
- Maximum genes per cell: 10,000.
- Maximum ribosomal RNA content: 80%.
- Mitochondrial content cutoff: 14.9%.

Cells not meeting these criteria were excluded from further analysis.

### scRNA-seq Normalization, Integration, and Clustering

After QC, the data were normalized using the standard normalization and scaling functions in Seurat version 5.1.0. To integrate multiple datasets and correct for batch effects, the Seurat anchor gene based canonical correlation analysis method was applied. The final dataset was composed of 99,596 cells. Cell clustering was performed using the Seurat version 5.1.0 package with the resolution parameter set to 0.6, resulting in 23 cell clusters spanning 4 stages of product development (days 0, 9, 14, and 25).

### scRNA-seq Pseudobulk Analysis

A pseudobulk analysis of scRNA-seq samples was performed to determine whether additional gene signatures influenced the responsiveness of samples at day 0. Seurat’s aggregate expression function produced a pseudobulk expression matrix from the single-cell data by aggregating the expression values of individual cells into sample-level group profiles. The resulting pseudobulk matrix represented samples from each process step. Pseudobulk counts representing the day 0 samples were inputted into a standard DESeq workflow using default size factor, dispersion, and negative binomial model fitting, in which the design matrix tests the factor of responsiveness. Differentially expressed genes were selected based on an adjusted *P* value less than 0.05. Differentially expressed genes associated with eliciting a clinical response or not eliciting a clinical response were separately tested for enrichment in Hallmark pathways using the enricher function from the clusterProfiler package. A hypergeometric test using an adjusted *P* value less than 0.05 was used to identify hallmark pathways enriched with differentially expressed genes.

### MTV Assessment

MTV analysis was performed on 26 patients who underwent 18F-fluorodeoxyglucose-PET/computed tomography (FDG-PET/CT) scans (PET-CT scans) at screening and first longitudinal scans during the dose escalation and expansion phase of the study. The preprocessing and analysis were done using the following steps in a validated pipeline and software (Invicro, LLC). All PET/CT scans underwent a series of initial preprocessing steps prior to tumor region of interest (ROI) delineation and quantitative PET analysis. Images were resampled to an isotropic voxel size of 4 mm to ensure consistency across sites and patients, and PET images were converted to units of standardized uptake value (SUV) using injected dose and patient weight information. Co-registration of PET images to anatomic CT scans was confirmed and adjusted as necessary.

Preprocessed PET/CT scans were initially reviewed by a trained image analyst to delineate ROIs for all FDG-avid lesions noted on PET scans. In cases of high bone lesion burden (>10 lesions), an automated deep learning algorithm was used to segment the entire skeleton and marrow space, and disease was defined as those voxels within this mask with an SUV greater than 1.5 times background red marrow uptake. A lumbar vertebra with no apparent disease was used to define background marrow uptake, with an alternative threshold of two times blood pool in an aorta ROI used in patients with extensive bone disease. All other lesions were delineated in three dimensions using a combination of semi-automated (isocontouring) and manual methods. Additionally, the following reference regions were created to assess background uptake levels in liver and blood: (i) a spherical reference region was placed in an unaffected liver region away from vessels; (ii) a vertically oriented, cylindrical reference region was placed in the descending thoracic aorta. Lesions were assigned an anatomic label according to predefined anatomic categories. The generated ROIs were reviewed by a qualified radiologist or nuclear medicine physician for accuracy in tumor delineation and placement of the reference regions and modified as needed.

For all lesions delineated with three-dimensional ROIs, the following metrics were generated: mean SUV (SUV_mean_), maximum SUV (SUV_max_), MTV, and total lesion glycolysis (TLG; product of SUV_mean_ and MTV), which is a metric describing total FDG uptake within each lesion. For each patient, the following summary-level metrics describing the aggregate of all identified lesions were generated for the whole body (all sites combined): whole-body SUV_mean_, whole-body maximum SUV_max_, whole-body MTV, whole-body TLG, and total number of lesions identified. The same aggregate measures were also computed for specific predefined disease sites. In addition, the background FDG uptake levels were calculated as the SUV_mean_ and SUV_max_ within the liver and blood pool ROIs, segmented as described above.

### Statistical Analyses and Study Population

All enrolled patients in this study were in the intent-to-treat (ITT) set. The safety analysis set and modified ITT set included patients who received TAK-007, which was used for safety and efficacy analyses to determine the RP2D. Patients who received TAK-007 and had sufficient data to estimate one or more CK parameters were part of the CK analysis set. The pharmacodynamic set included patients from the safety analysis set who had baseline and at least one post-baseline sample assessment.

ORR and best response by the investigator assessment were calculated by the dose level in the escalation cohorts and for all patients in the expansion cohorts with exact 2-sided 95% CIs. DOR for responders was defined as the time from the date of the first documented objective response to the date of the first documented disease progression or death. Patients not meeting the criteria for disease progression or death, or initiating new anticancer therapy, were censored at the last disease assessment. PFS and OS, including medians and two-sided 95% CIs, were estimated using the Kaplan–Meier method.

The association between CK parameters and response was evaluated using a logistic regression model (generalized linear model with a binomial distribution and logit link), assessing the relationship between C_max_ (copies/μg) and the probability of complete response (CR versus PD/SD/PR). Corresponding *P* values were derived using a Wald test.

## Supplementary Material

Table S1 Supplementary Table 1 shows the representativeness of study patients.

Table S2 Supplementary Table 2 shows the summary of patients with detectable anti-HLA and anti-CAR antibodies across dose escalation, 800M dose expansion and across all patients.

Table S3 Supplementary Table 3 shows the categorization of drug product lots based on clinical responses

Figure S1 Supplementary Figure S1 shows the metabolic tumor volume analysis conducted in all patients who received 800M TAK-007, patients with LBCL who received TAK-007 800M, and in patients iNHL who received TAK-007 800M

Figure S2 Supplementary Figure S2 shows the exposure-safety relationship, with logistic regression modeling the probability of adverse events in the safety population.

Figure S3 Supplementary Figure S3 shows B-cell counts over time in the pharmacodynamic population during dose escalation and dose expansion.

Figure S4 Supplementary Figure S4 shows Interleukin-15, Interleukin-6, Interferon gamma, tumor necrosis factor, granulocyte macrophage colony stimulating factor, interleukin- 8, interleukin-10, interleukin-12 profiles following TAK-007 administration.

Figure S5 Supplementary Figure S5 shows differential gene expression in Day 0 samples that did versus did not elicit a clinical response, based on pseudobulk analysis.

Figure S6 Supplementary Figure S6 shows in vitro and in vivo characterization of the cryopreserved and fresh products. (A) In vitro cytotoxicity comparing fresh controls with cryopreserved cells harvested at different timepoints, showing improved activity in later-harvested products across four donors. (B) Comparable *in vitro* cytotoxicity of cells harvested on Days 18 and 25 using semi-automated or manual manufacturing processes (1,8). (C) *In vivo* anti-tumor efficacy demonstrating similar survival benefit between optimized cryopreserved products and fresh controls following uAPC restimulation. (D) Similarity analysis of fresh CD19 CAR-NK products and TAK-007 cryoproducts across key quality attributes, including viability, NK purity, CAR expression, VCN/CAR+, and cytotoxicity.

## Data Availability

The datasets, including the redacted study protocols, redacted statistical analysis plans, and individual participant data supporting the results of the completed study, will be made available after the publication of this article within 3 months from the initial request. The data will be provided after its deidentification, in compliance with applicable privacy laws, data protection, and requirements for consent. Meanwhile, the read counts are available at https://zenodo.org/records/19615830.

## References

[1] Kite Pharma, Inc . Yescarta (axicabtagene ciloleucel) prescribing information. Santa Monica (CA): Kite Pharma, Inc.; 2021 [cited 2025 Oct 20]. Available from: https://www.gilead.com/-/media/files/pdfs/medicines/oncology/yescarta/yescarta-pi.pdf.

[2] Novartis Pharmaceutical Corporation . Kymriah (tisagenlecleucel) prescribing information. 2021 [cited 2025 Oct 20]. Available from: https://www.novartis.com/us-en/sites/novartis_us/files/kymriah.pdf.

[3] Kite Pharma, Inc . Tecartus (brexucabtagene autoleucel) prescribing information. Santa Monica (CA): Kite Pharma, Inc.; 2021 [cited 2025 Oct 20]. Available from: https://www.gilead.com/-/media/files/pdfs/medicines/oncology/tecartus/tecartus-pi.pdf.

[4] Juno Therapeutics Inc . Breyanzi (lisocabtagene maraleucel) prescribing information. 2021 [cited 2025 Oct 20]. Available from: https://packageinserts.bms.com/pi/pi_breyanzi.pdf.

[5] Gajra A , Zalenski A, Sannareddy A, Jeune-Smith Y, Kapinos K, Kansagra A. Barriers to chimeric antigen receptor T-cell (CAR-T) therapies in clinical practice. Pharmaceut Med 2022;36:163–71. 10.1007/s40290-022-00428-w. PMC9217916 35672571

[6] Rafiq S , Hackett CS, Brentjens RJ. Engineering strategies to overcome the current roadblocks in CAR T cell therapy. Nat Rev Clin Oncol 2020;17:147–67. 10.1038/s41571-019-0297-y. PMC7223338 31848460

[7] Mitra A , Barua A, Huang L, Ganguly S, Feng Q, He B. From bench to bedside: the history and progress of CAR T cell therapy. Front Immunol 2023;14:1188049. 10.3389/fimmu.2023.1188049. PMC10225594 37256141

[8] Brudno JN , Kochenderfer JN. Recent advances in CAR T-cell toxicity: mechanisms, manifestations and management. Blood Rev 2019;34:45–55. 10.1016/j.blre.2018.11.002. PMC6628697 30528964

[9] Lonez C , Breman E. Allogeneic CAR-T therapy technologies: has the promise been met? Cells 2024;13:146. 10.3390/cells13020146. PMC10814647 38247837

[10] Cappell KM , Kochenderfer JN. Long-term outcomes following CAR T cell therapy: what we know so far. Nat Rev Clin Oncol 2023;20:359–71. 10.1038/s41571-023-00754-1. PMC10100620 37055515

[11] Basar R , Daher M, Rezvani K. Next-generation cell therapies: the emerging role of CAR-NK cells. Blood Adv 2020;4:5868–76. 10.1182/bloodadvances.2020002547. PMC7686910 33232480

[12] Liu E , Tong Y, Dotti G, Shaim H, Savoldo B, Mukherjee M, . Cord blood NK cells engineered to express IL-15 and a CD19-targeted CAR show long-term persistence and potent antitumor activity. Leukemia 2018;32:520–31. 10.1038/leu.2017.226. PMC6063081 28725044

[13] Wang W , Liu Y, He Z, Li L, Liu S, Jiang M, . Breakthrough of solid tumor treatment: CAR-NK immunotherapy. Cell Death Discov 2024;10:40. 10.1038/s41420-024-01815-9. PMC10799930 38245520

[14] Marin D , Li Y, Basar R, Rafei H, Daher M, Dou J, . Safety, efficacy and determinants of response of allogeneic CD19-specific CAR-NK cells in CD19+ B cell tumors: a phase 1/2 trial. Nat Med 2024;30:772–84. 10.1038/s41591-023-02785-8. PMC10957466 38238616

[15] Liu E , Marin D, Banerjee P, Macapinlac HA, Thompson P, Basar R, . Use of CAR-transduced natural killer cells in CD19-positive lymphoid tumors. N Engl J Med 2020;382:545–53. 10.1056/NEJMoa1910607. PMC7101242 32023374

[16] Stuart T , Butler A, Hoffman P, Hafemeister C, Papalexi E, Mauck WM 3rd, . Comprehensive integration of single-cell data. Cell 2019;177:1888–902.e21. 10.1016/j.cell.2019.05.031. PMC6687398 31178118

[17] Hao Y , Hao S, Andersen-Nissen E, Mauck WM 3rd, Zheng S, Butler A, . Integrated analysis of multimodal single-cell data. Cell 2021;184:3573–87.e29. 10.1016/j.cell.2021.04.048. PMC8238499 34062119

[18] Monick S , Rosenthal A. Circulating tumor DNA as a complementary prognostic biomarker during CAR-T therapy in B-cell non-Hodgkin lymphomas. Cancers (Basel) 2024;16:1881. 10.3390/cancers16101881. PMC11120115 38791959

[19] Frank MJ , Hossain NM, Bukhari A, Dean E, Spiegel JY, Claire GK, . Monitoring of circulating tumor DNA improves early relapse detection after axicabtagene ciloleucel infusion in large B-cell lymphoma: results of a prospective multi-institutional trial. J Clin Oncol 2021;39:3034–43. 10.1200/JCO.21.00377. PMC10166351 34133196

[20] Foerster AK , Kronenthaler C, Ranganathan L, Bandara S, Woestmann C, Yaung S, . Early prediction of treatment response by circulating tumor DNA profiling in patients with diffuse large B-cell lymphoma receiving CAR T-cell therapy. Blood 2023;142:2987. 10.1182/blood-2023-178871.

[21] Zou H , Liu W, Wang X, Wang Y, Wang C, Qiu C, . Dynamic monitoring of circulating tumor DNA reveals outcomes and genomic alterations in patients with relapsed or refractory large B-cell lymphoma undergoing CAR T-cell therapy. J Immunother Cancer 2024;12:e008450. 10.1136/jitc-2023-008450. PMC11146396 38443094

[22] Fang F , Xie S, Chen M, Li Y, Yue J, Ma J, . Advances in NK cell production. Cell Mol Immunol 2022;19:460–81. 10.1038/s41423-021-00808-3. PMC8975878 34983953

[23] Bachanova V , Ghobadi A, Patel K, Park JH, Flinn IW, Shah P, . Safety and efficacy of FT596, a first-in-class, multi-antigen targeted, off-the-shelf, iPSC-derived CD19 CAR NK cell therapy in relapsed/refractory B-cell lymphoma. Blood 2021;138:823. 10.1182/blood-2021-151185.

[24] Islam R , Pupovac A, Evtimov V, Boyd N, Shu R, Boyd R, . Enhancing a natural killer: modification of NK cells for cancer immunotherapy. Cells 2021;10:1058. 10.3390/cells10051058. PMC8146003 33946954

[25] Nieto Y , Banerjee P, Kaur I, Basar R, Li Y, Daher M, . Allogeneic NK cells with a bispecific innate cell engager in refractory relapsed lymphoma: a phase 1 trial. Nat Med 2025;31:1987–93. 10.1038/s41591-025-03640-8. PMC13218020 40186077

[26] Liu S , Yuan Y. Bayesian optimal interval designs for phase I clinical trials. J R Stat Soc Ser C Appl Stat 2015;64:507–23. 10.1111/rssc.12089.

[27] Yuan Y , Hess KR, Hilsenbeck SG, Gilbert MR. Bayesian optimal interval design: a simple and well-performing design for phase I oncology trials. Clin Cancer Res 2016;22:4291–301. 10.1158/1078-0432.CCR-16-0592. PMC5047439 27407096

[28] Zhou H , Yuan Y, Nie L. Accuracy, safety, and reliability of novel phase I trial designs. Clin Cancer Res 2018;24:4357–64. 10.1158/1078-0432.CCR-18-0168. 29661774

[29] ClinicalTrials.gov . A study of TAK-007 in adults with relapsed or refractory (r/r) B-cell non-Hodgkin lymphoma (NHL). ClinicalTrials.gov ID NCT05020015. Bethesda (MD): National Library of Medicine; 2026 [cited 2026 Apr 14]. Available from: https://clinicaltrials.gov/study/NCT05020015.

[30] NCI . Common Terminology Criteria for Adverse Events (CTCAE) Version 5.0. U.S. Department of Health and Human Services, National Institutes of Health. Bethesda (MD): National Cancer Institute; 2017 [cited 2025 Oct 20]. Available from: https://dctd.cancer.gov/research/ctep-trials/for-sites/adverse-events/ctcae-v5-5x7.pdf.

[31] Lee DW , Santomasso BD, Locke FL, Ghobadi A, Turtle CJ, Brudno JN, . ASTCT consensus grading for cytokine release syndrome and neurologic toxicity associated with immune effector cells. Biol Blood Marrow Transpl 2019;25:625–38. 10.1016/j.bbmt.2018.12.758. PMC12180426 30592986

[32] Harris AC , Young R, Devine S, Hogan WJ, Ayuk F, Bunworasate U, . International, multicenter standardization of acute graft-versus-host disease clinical data collection: a report from the Mount Sinai Acute GVHD International Consortium. Biol Blood Marrow Transpl 2016;22:4–10. 10.1016/j.bbmt.2015.09.001. PMC4706482 26386318

[33] Jagasia MH , Greinix HT, Arora M, Williams KM, Wolff D, Cowen EW, . National Institutes of Health consensus development project on criteria for clinical trials in chronic graft-versus-host disease: I. The 2014 Diagnosis and Staging Working Group report. Biol Blood Marrow Transpl 2015;21:389–401.e1. 10.1016/j.bbmt.2014.12.001. PMC4329079 25529383

[34] Cheson BD , Fisher RI, Barrington SF, Cavalli F, Schwartz LH, Zucca E, . Recommendations for initial evaluation, staging, and response assessment of Hodgkin and non-Hodgkin lymphoma: the Lugano classification. J Clin Oncol 2014;32:3059–68. 10.1200/JCO.2013.54.8800. PMC4979083 25113753

[35] Klimova N , Close S, Kurtz DM, Hockett RD, Hyland L. Analytical validation of a circulating tumor DNA assay using PhasED-Seq technology for detecting residual disease in B-cell malignancies. Oncotarget 2025;16:329–36. 10.18632/oncotarget.28719. PMC12068320 40353795

[36] DeTora LM , Toroser D, Sykes A, Vanderlinden C, Plunkett FJ, Lane T, . Good Publication Practice (GPP) guidelines for company-sponsored biomedical research: 2022 update. Ann Intern Med 2022;175:1298–304. 10.7326/M22-1460. 36037471

