# Supplementary material for: A Phase II, Open-label, Multicenter Study of the Safety and Efficacy of TAK-007 in Adult Patients with Relapsed/Refractory B-cell Non–Hodgkin Lymphoma
Source: Blood Cancer Discov. 2026 Apr 17;7(6):892–908. doi: 10.1158/2643-3230.BCD-25-0323 (PMC13728790; doi:10.1158/2643-3230.BCD-25-0323)
Supplement: Supplementary file 1 [file bcd-25-0323_figure_s1_suppsf1.pdf]

Supplementary Figure 1. Metabolic tumor volume analysis conducted in the safety population.

A.

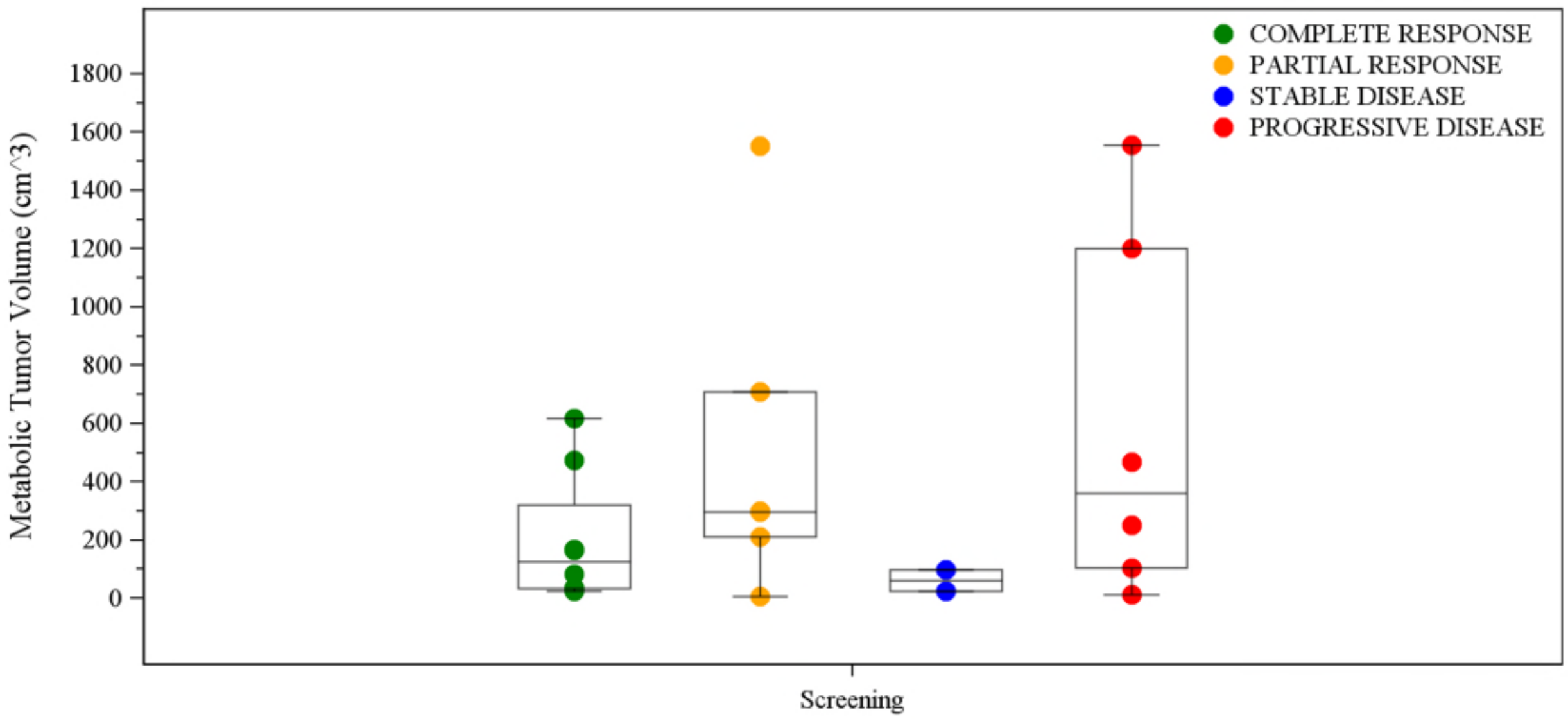

B.

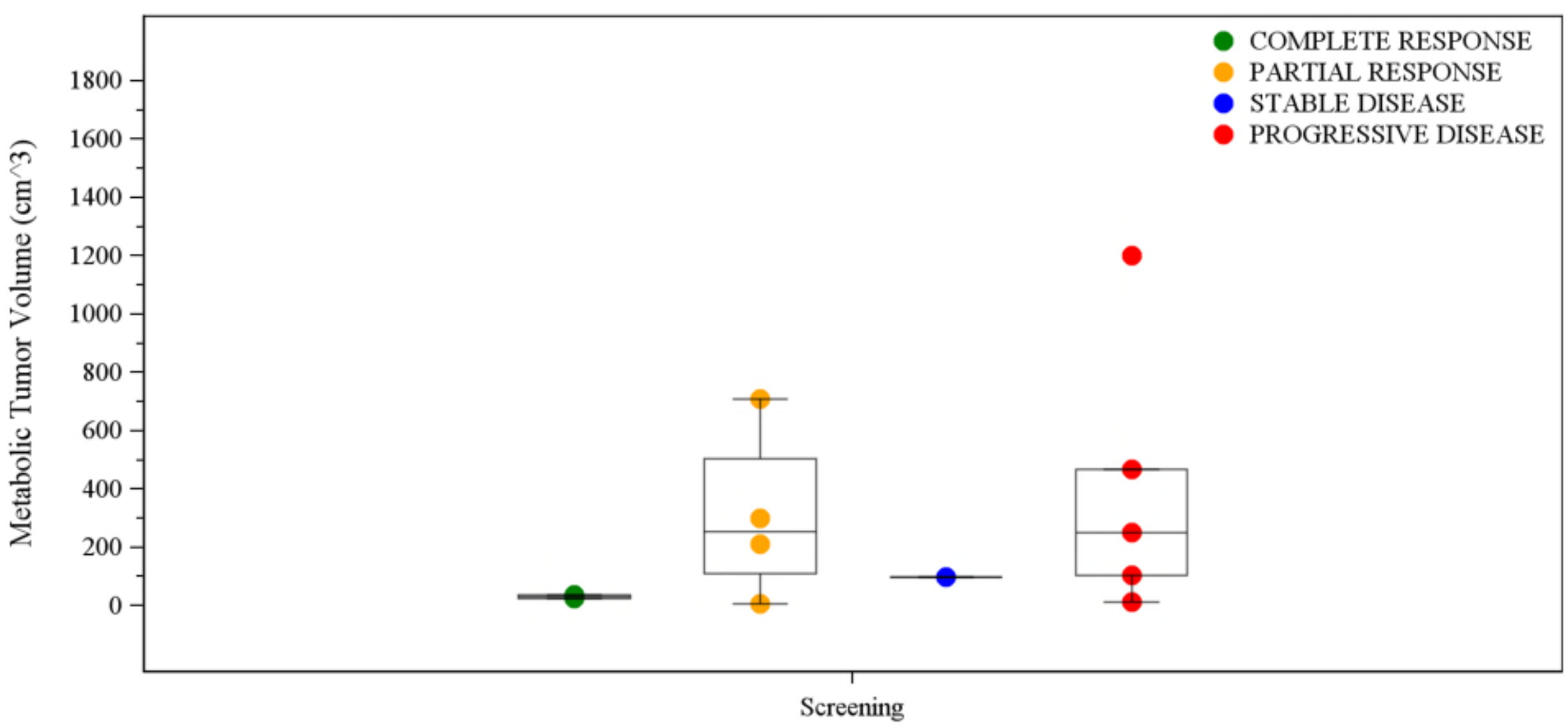

C.

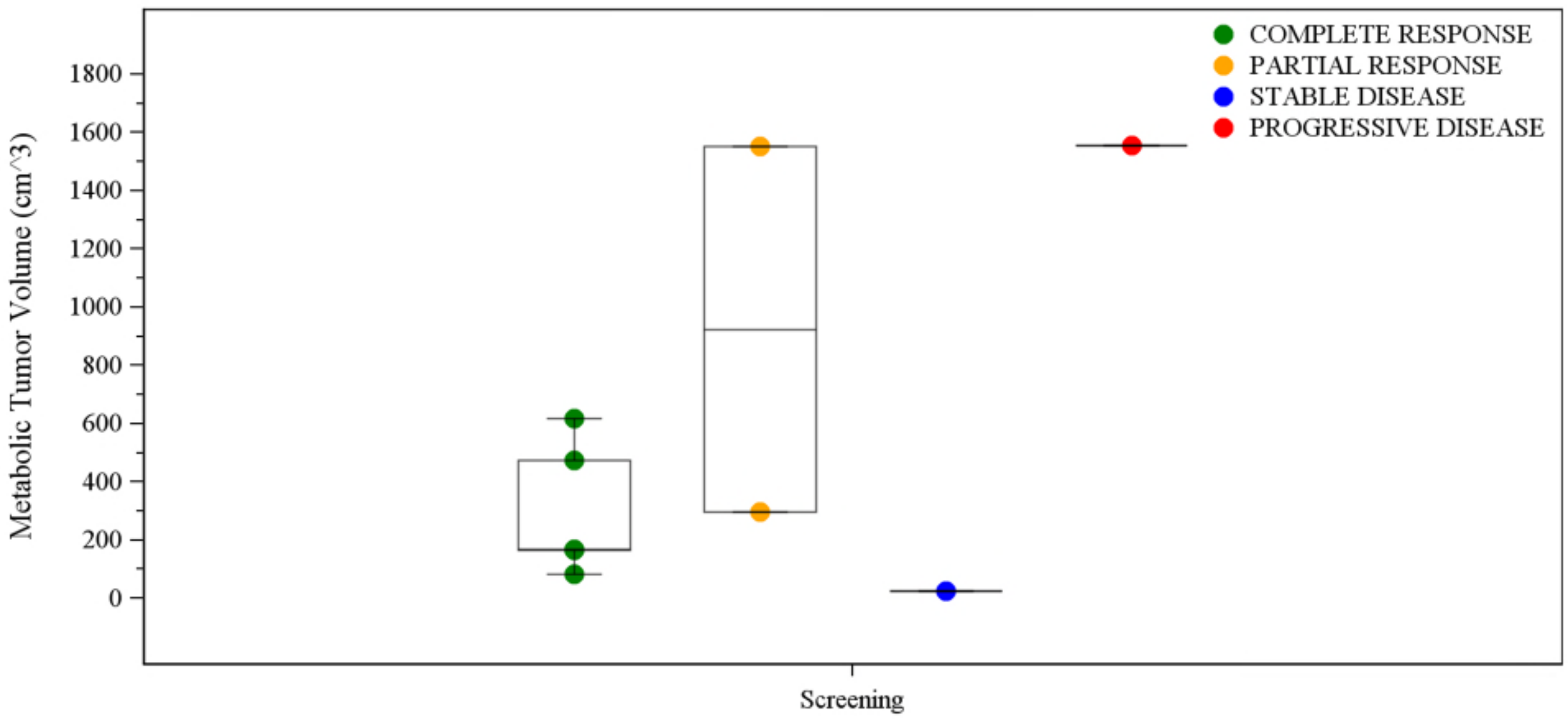

A. Metabolic tumor volume at screening in all patients who received TAK-007 800M, B. Metabolic tumor volume with LBCL who received TAK-007 800M; C. Metabolic tumor volume in patients with iNHL who received TAK-007 800M.

iNHL, indolent non-Hodgkin Lymphoma; LBCL, large B-cell lymphoma.
