# Supplementary material for: A Phase II, Open-label, Multicenter Study of the Safety and Efficacy of TAK-007 in Adult Patients with Relapsed/Refractory B-cell Non–Hodgkin Lymphoma
Source: Blood Cancer Discov. 2026 Apr 17;7(6):892–908. doi: 10.1158/2643-3230.BCD-25-0323 (PMC13728790; doi:10.1158/2643-3230.BCD-25-0323)
Supplement: Supplementary file 2 [file bcd-25-0323_figure_s2_suppsf2.pdf]

Supplementary Figure 2. Exposure-safety relationship – logistic regression of the probability of observing adverse events in the safety population.

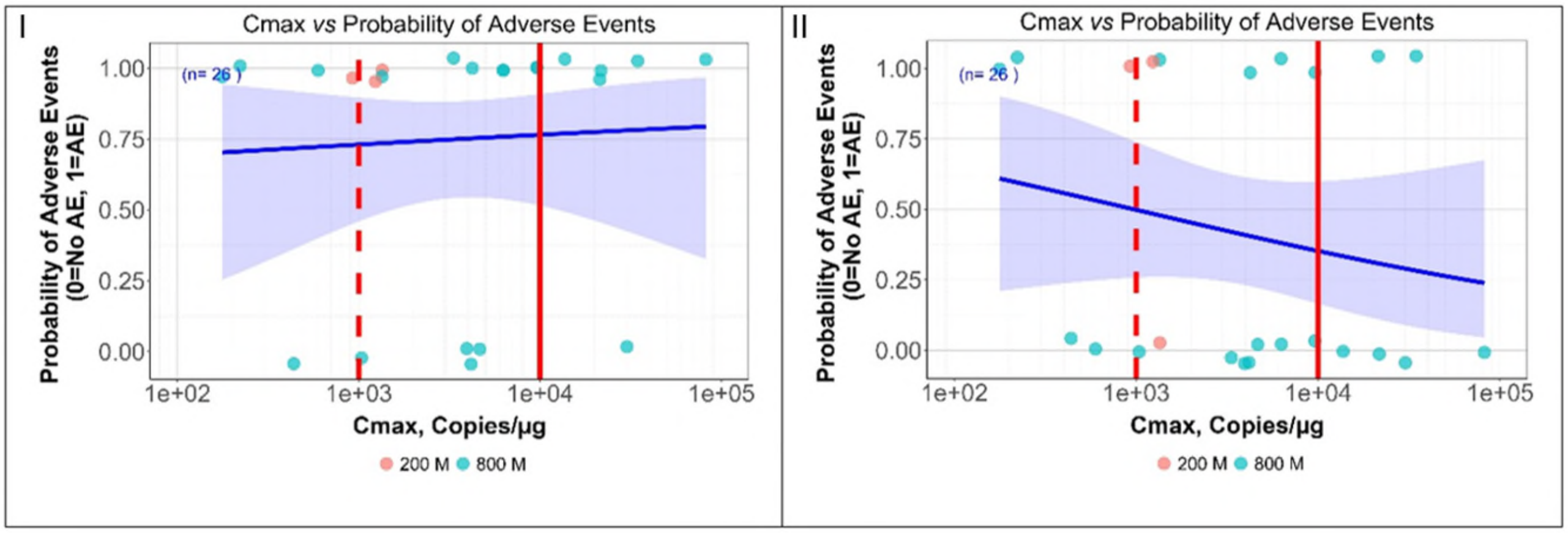

Dark blue line indicates the predicted probability of AEs and light blue ribbon indicates 95% CI of predicted probability of AEs. Individual C_{max} values are shown as colored dots.

0, no adverse event; 1, ≤ grade 4 adverse event; AE, adverse events; CI, confidence interval; C_{max}, maximum serum concentration.
