# Supplementary material for: A Phase II, Open-label, Multicenter Study of the Safety and Efficacy of TAK-007 in Adult Patients with Relapsed/Refractory B-cell Non–Hodgkin Lymphoma
Source: Blood Cancer Discov. 2026 Apr 17;7(6):892–908. doi: 10.1158/2643-3230.BCD-25-0323 (PMC13728790; doi:10.1158/2643-3230.BCD-25-0323)
Supplement: Supplementary file 3 [file bcd-25-0323_figure_s3_suppsf3.pdf]

Supplementary Figure 3. B-cell counts over time in the pharmacodynamic population.

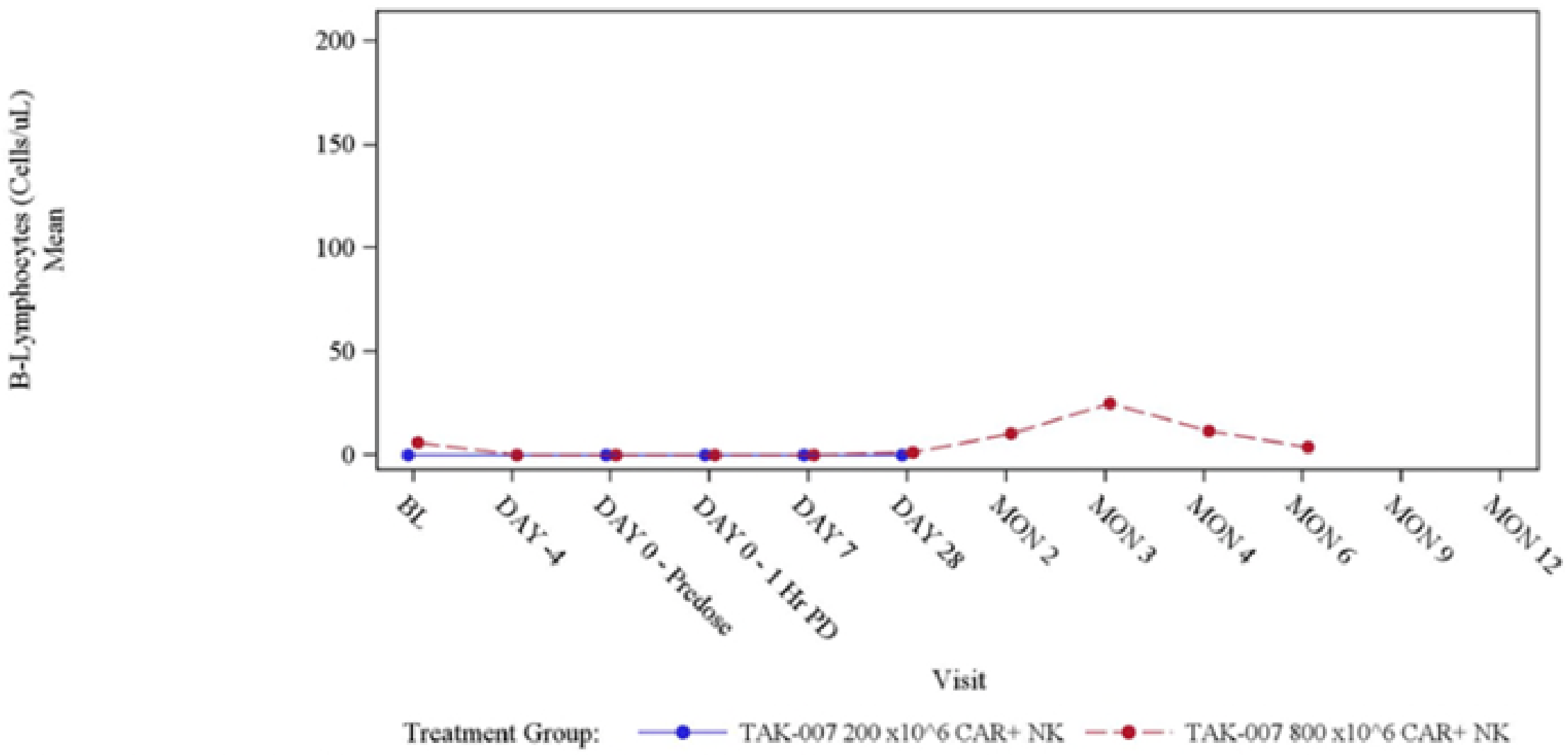

	BL	DAY -4	DAY 0 - Predose	DAY 0 - 1 Hr PD	DAY 7	DAY 28	MON 2	MON 3	MON 4	MON 6	MON 9	MON 12
TAK-007 200 x10^6 CAR+ NK	3	0	3	3	3	1	0	0	0	0	0	0
TAK-007 800 x10^6 CAR+ NK	5	1	5	5	4	5	2	1	1	1	0	0

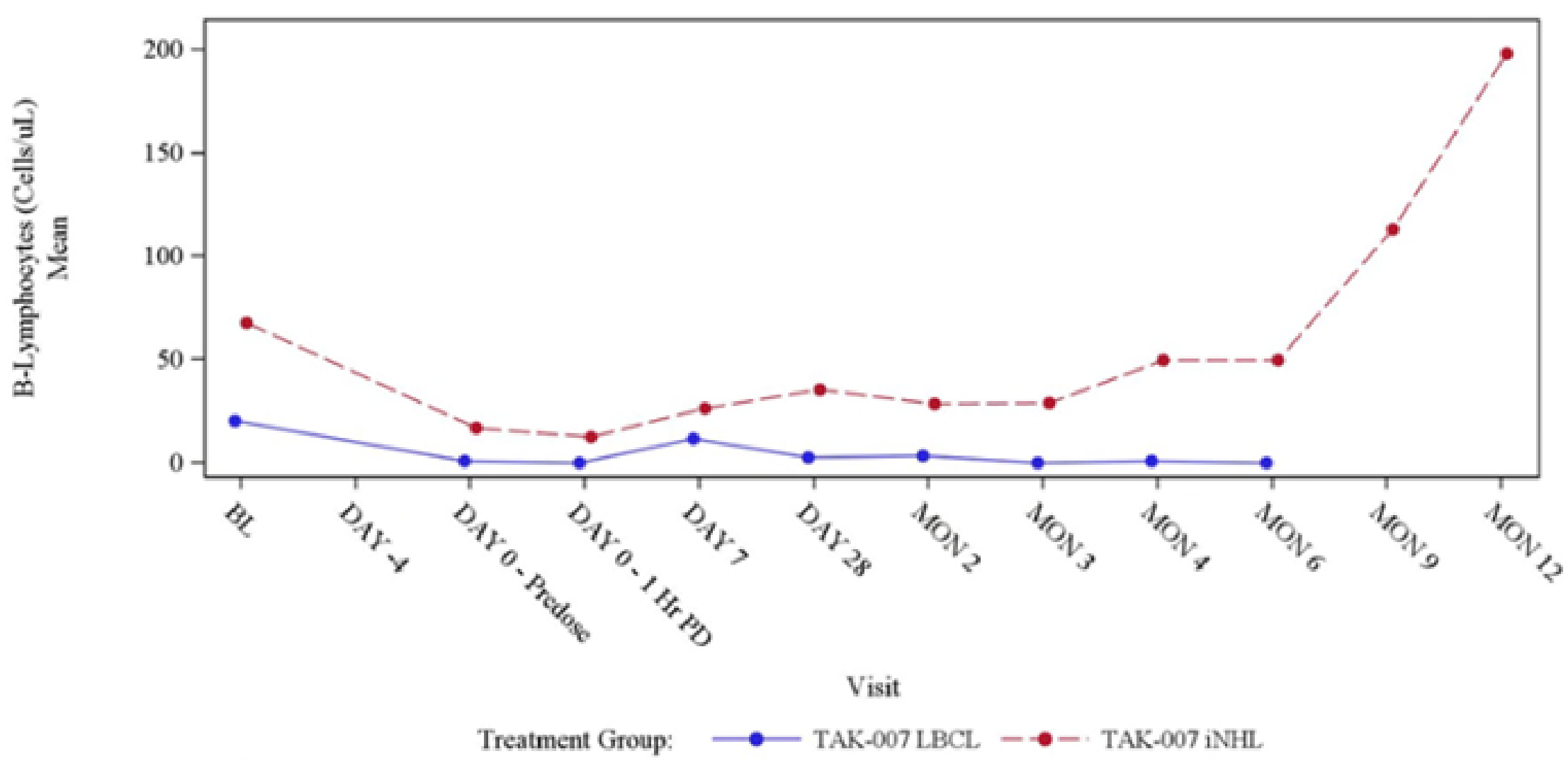

	BL	DAY -4	DAY 0 - Predose	DAY 0 - 1 Hr PD	DAY 7	DAY 28	MON 2	MON 3	MON 4	MON 6	MON 9	MON 12
TAK-007 LBCL	9	0	9	9	9	8	5	2	1	1	0	0
TAK-007 iNHL	6	0	5	7	5	6	6	6	4	5	2	1

BL, baseline; iNHL, indolent non-Hodgkin Lymphoma; LBCL, large B-cell lymphoma; MON, month; PD, progressive disease.
