# Supplementary material for: A Phase II, Open-label, Multicenter Study of the Safety and Efficacy of TAK-007 in Adult Patients with Relapsed/Refractory B-cell Non–Hodgkin Lymphoma
Source: Blood Cancer Discov. 2026 Apr 17;7(6):892–908. doi: 10.1158/2643-3230.BCD-25-0323 (PMC13728790; doi:10.1158/2643-3230.BCD-25-0323)
Supplement: Supplementary file 4 [file bcd-25-0323_figure_s4_suppsf4.pdf]

Supplementary Figure 4. Cytokine profiles following TAK-007 administration.

A.

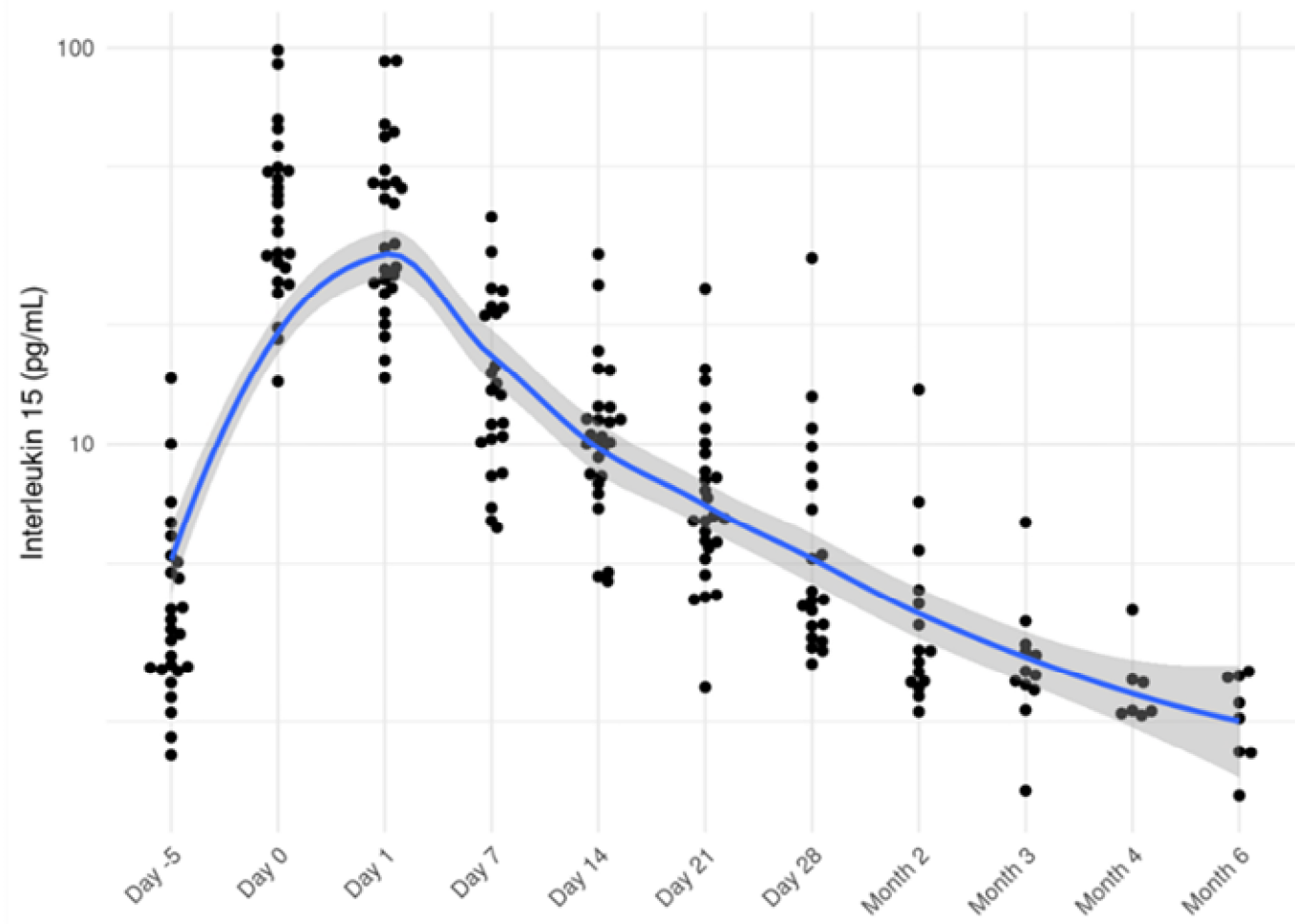

B.

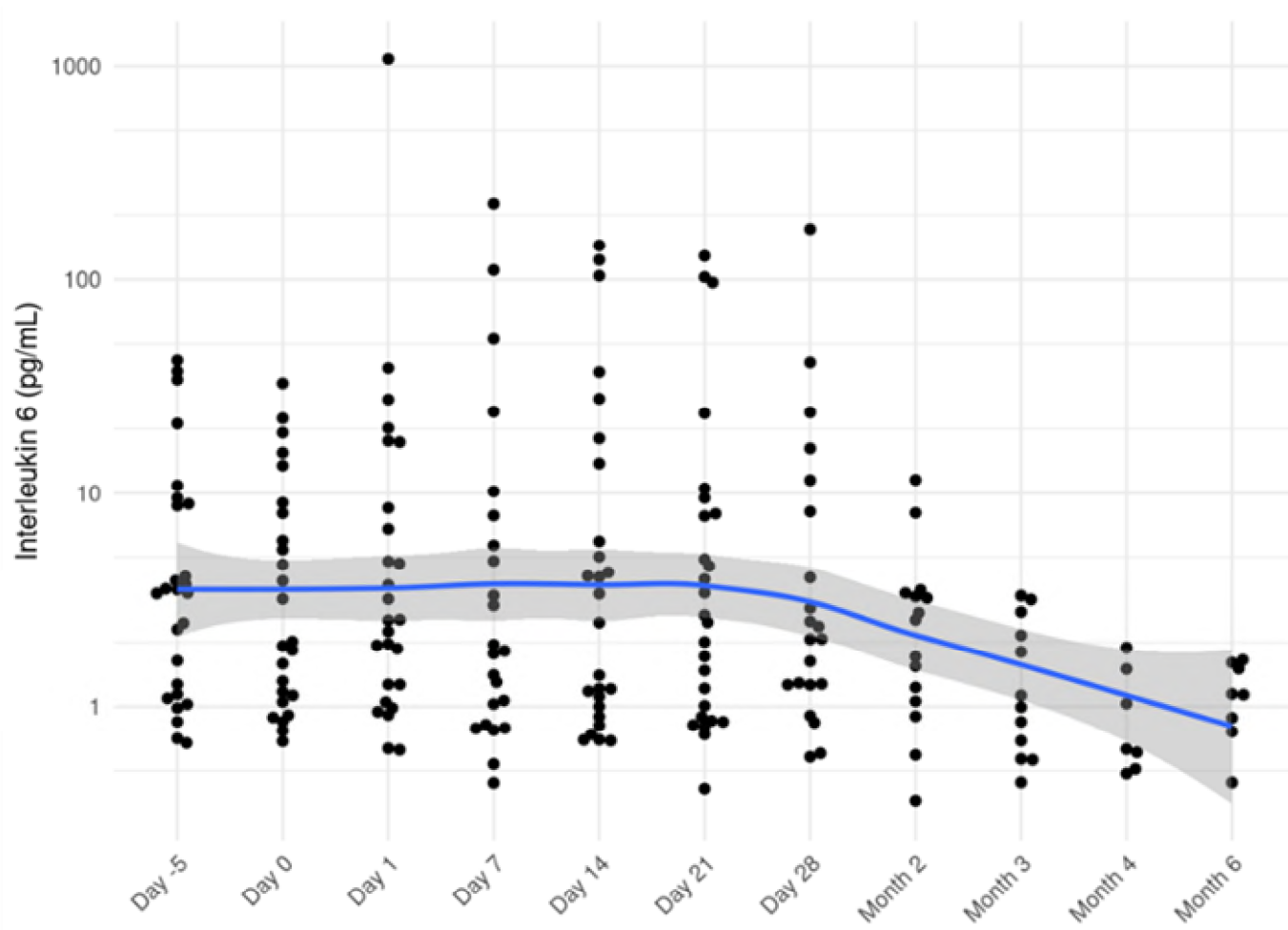

C.

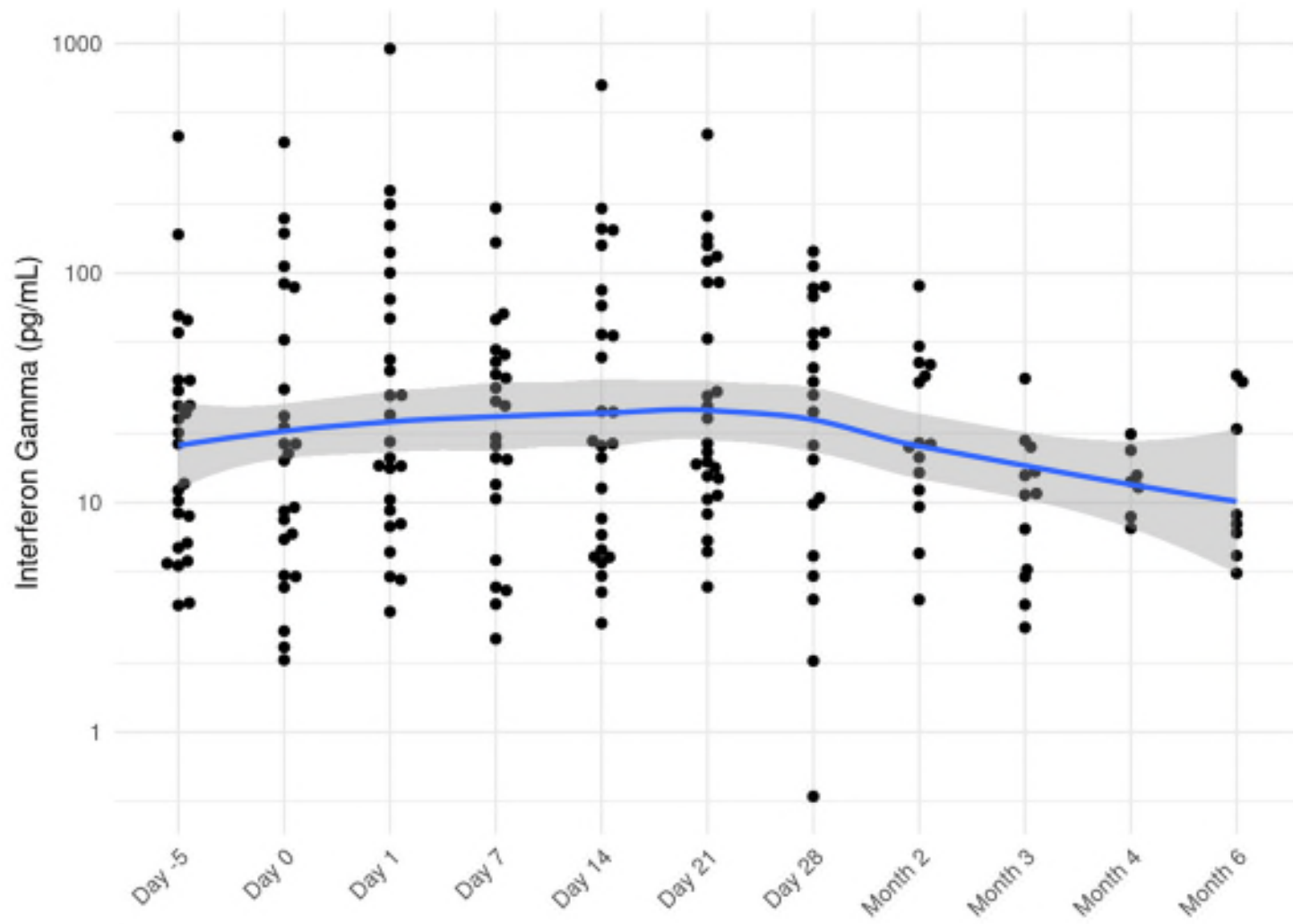
Interferon Gamma (pg/mL)
1000
100
10
1
Day -5
Day 0
Day 1
Day 7
Day 14
Day 21
Day 28
Month 2
Month 3
Month 4
Month 6

D.

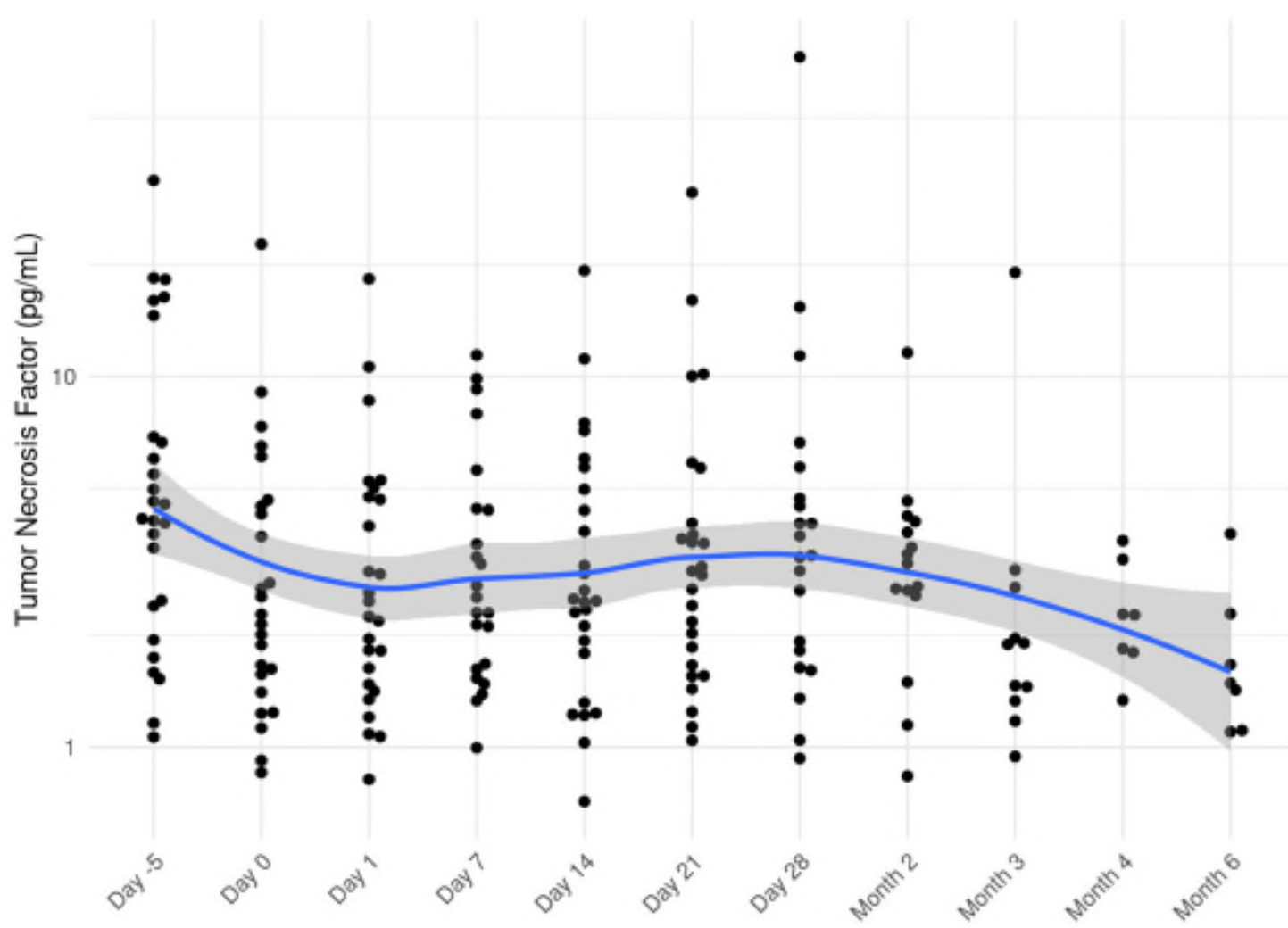
Tumor Necrosis Factor (pg/mL)
10
1
Day -5
Day 0
Day 1
Day 7
Day 14
Day 21
Day 28
Month 2
Month 3
Month 4
Month 6

E.

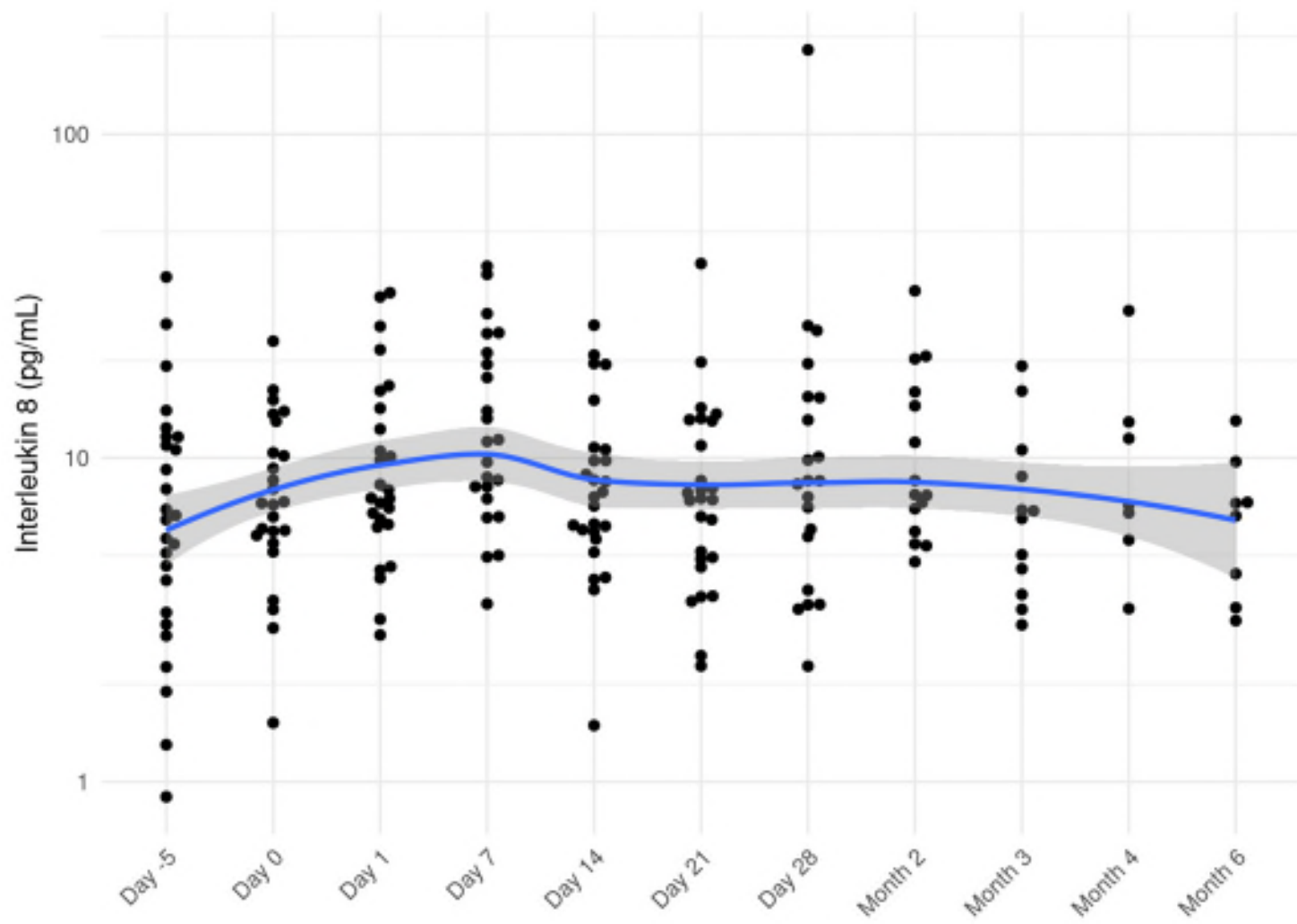
Interleukin 8 (pg/mL)
100
10
1
Day -5
Day 0
Day 1
Day 7
Day 14
Day 21
Day 28
Month 2
Month 3
Month 4
Month 6

F.

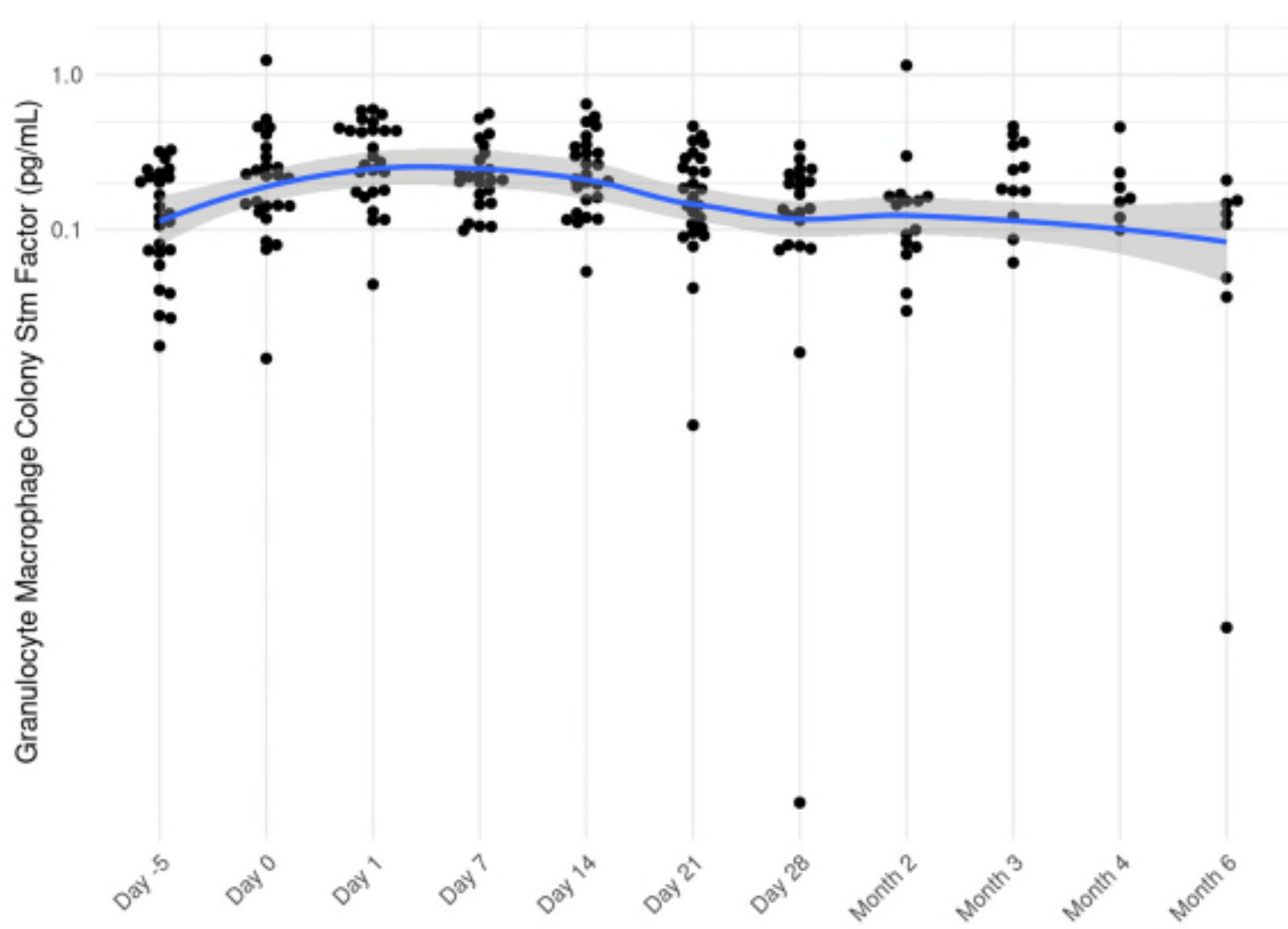
Granulocyte Macrophage Colony Stm Factor (pg/mL)
1.0
0.1
Day -5
Day 0
Day 1
Day 7
Day 14
Day 21
Day 28
Month 2
Month 3
Month 4
Month 6

G.

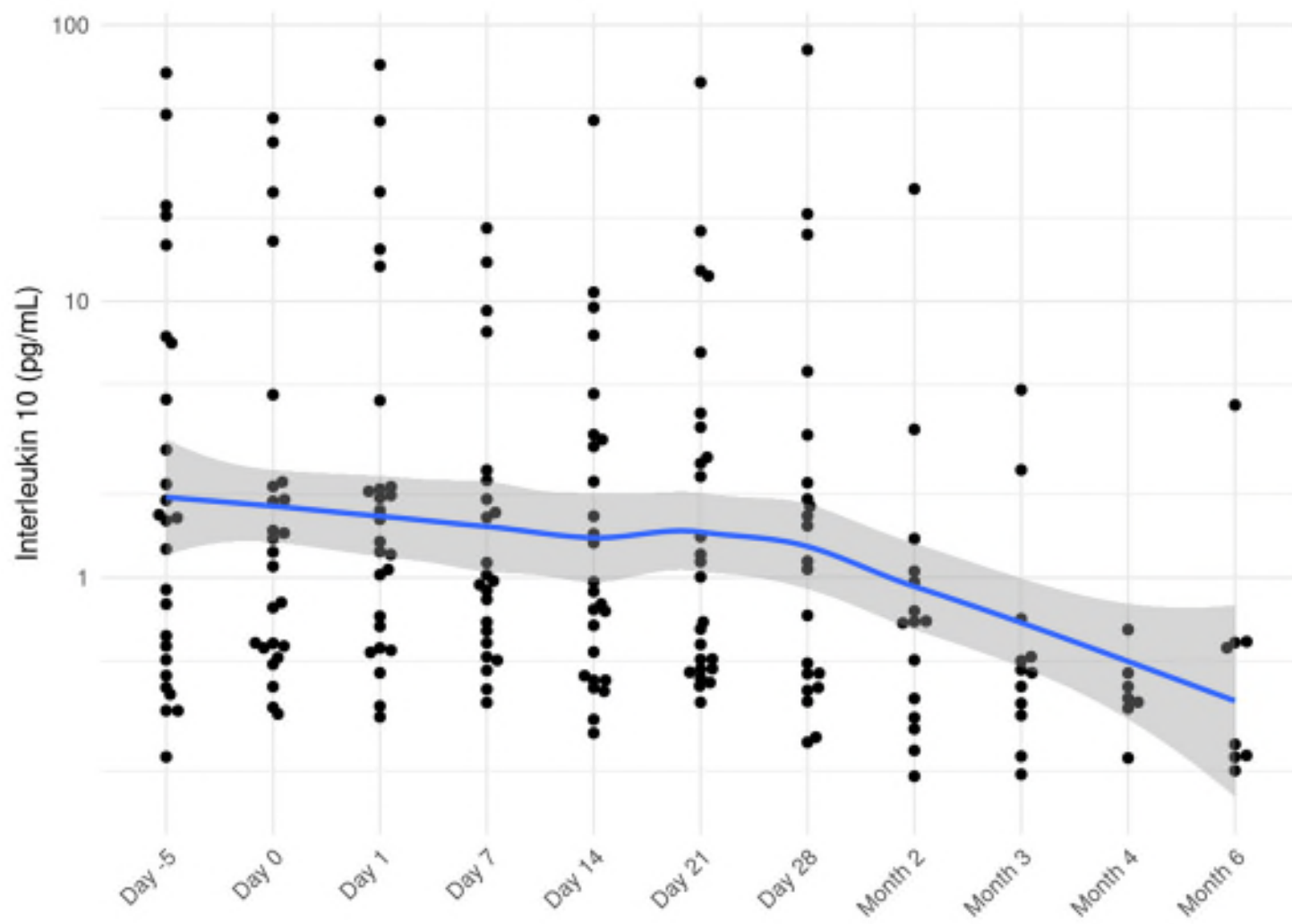

H.

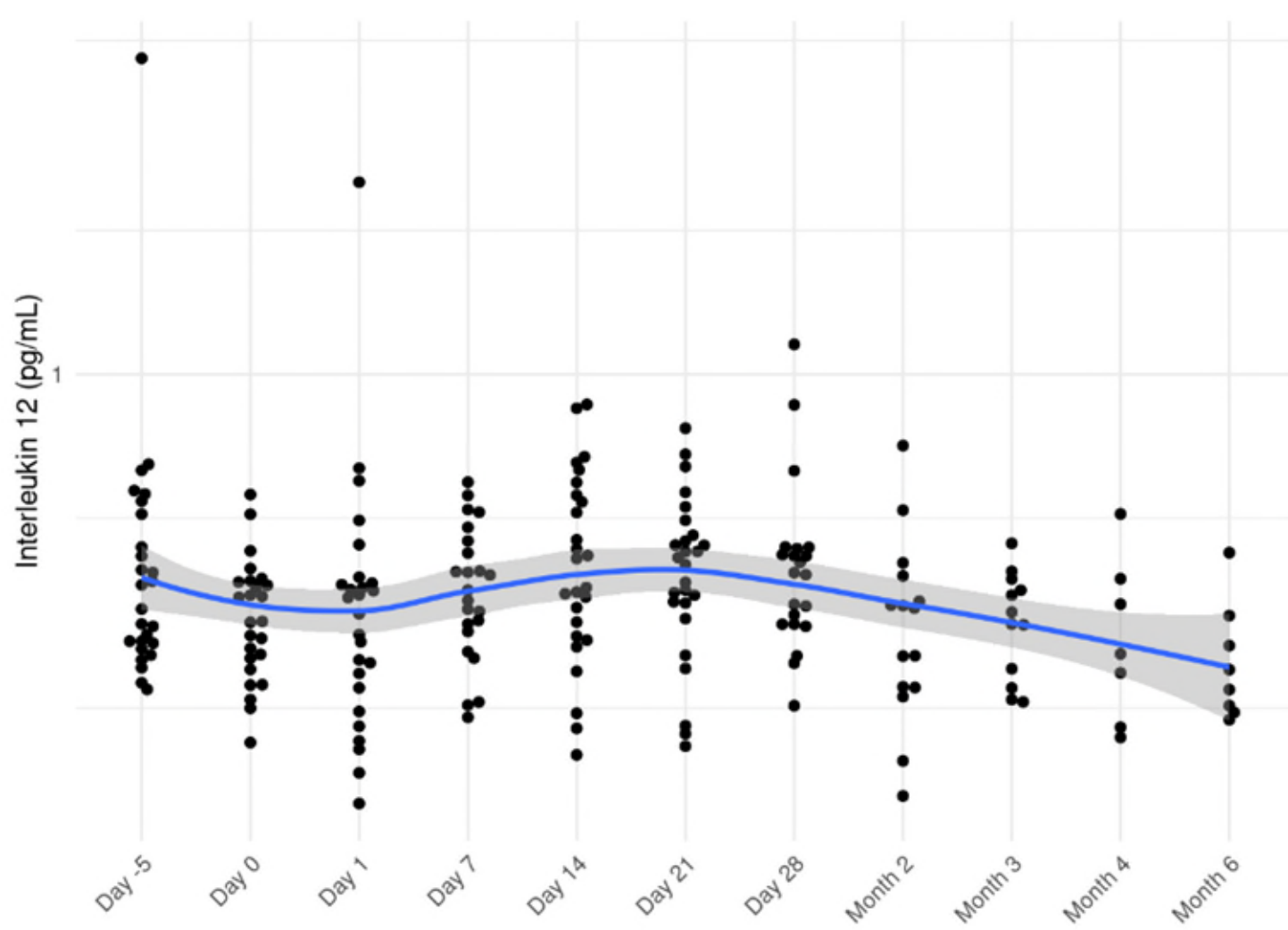

A. Interleukin-15; B. Interleukin-6, C. Interferon gamma; D. Tumor necrosis factor; E. Granulocyte macrophage colony stimulating factor; F. Interleukin-8; G. Interleukin-10; H. Interleukin-12 profiles following TAK-007 administration.

The blue lines with gray shading are LOESS curves (nonparametric regression) along with the standard error estimate. Note that this regression is done on the log10 scale as shown on the y-axis of the plots.
