# Supplementary material for: A Phase II, Open-label, Multicenter Study of the Safety and Efficacy of TAK-007 in Adult Patients with Relapsed/Refractory B-cell Non–Hodgkin Lymphoma
Source: Blood Cancer Discov. 2026 Apr 17;7(6):892–908. doi: 10.1158/2643-3230.BCD-25-0323 (PMC13728790; doi:10.1158/2643-3230.BCD-25-0323)

Supplementary Figure 5. Differential expression of genes in Day 0 samples that did vs. did not elicit a clinical response (Pseudobulk analysis)

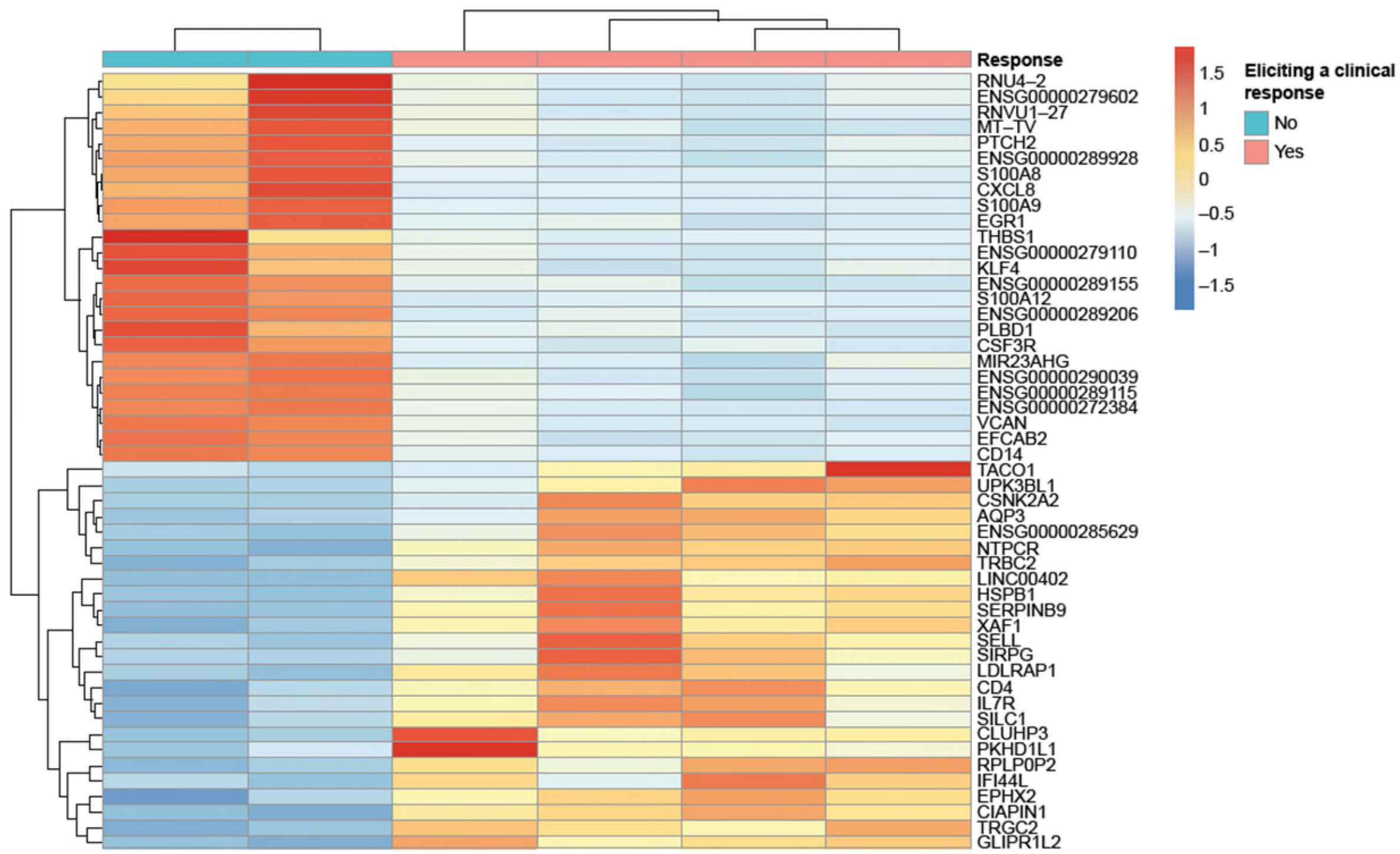

Supplement: Supplementary file 5 [file bcd-25-0323_figure_s5_suppsf5.pdf]
