# Supplementary material for: A Phase II, Open-label, Multicenter Study of the Safety and Efficacy of TAK-007 in Adult Patients with Relapsed/Refractory B-cell Non–Hodgkin Lymphoma
Source: Blood Cancer Discov. 2026 Apr 17;7(6):892–908. doi: 10.1158/2643-3230.BCD-25-0323 (PMC13728790; doi:10.1158/2643-3230.BCD-25-0323)
Supplement: Supplementary file 6 [file bcd-25-0323_figure_s6_suppsf6.pdf]

Supplementary Figure 6. In vitro and In vivo characterization of cryopreserved product and fresh product

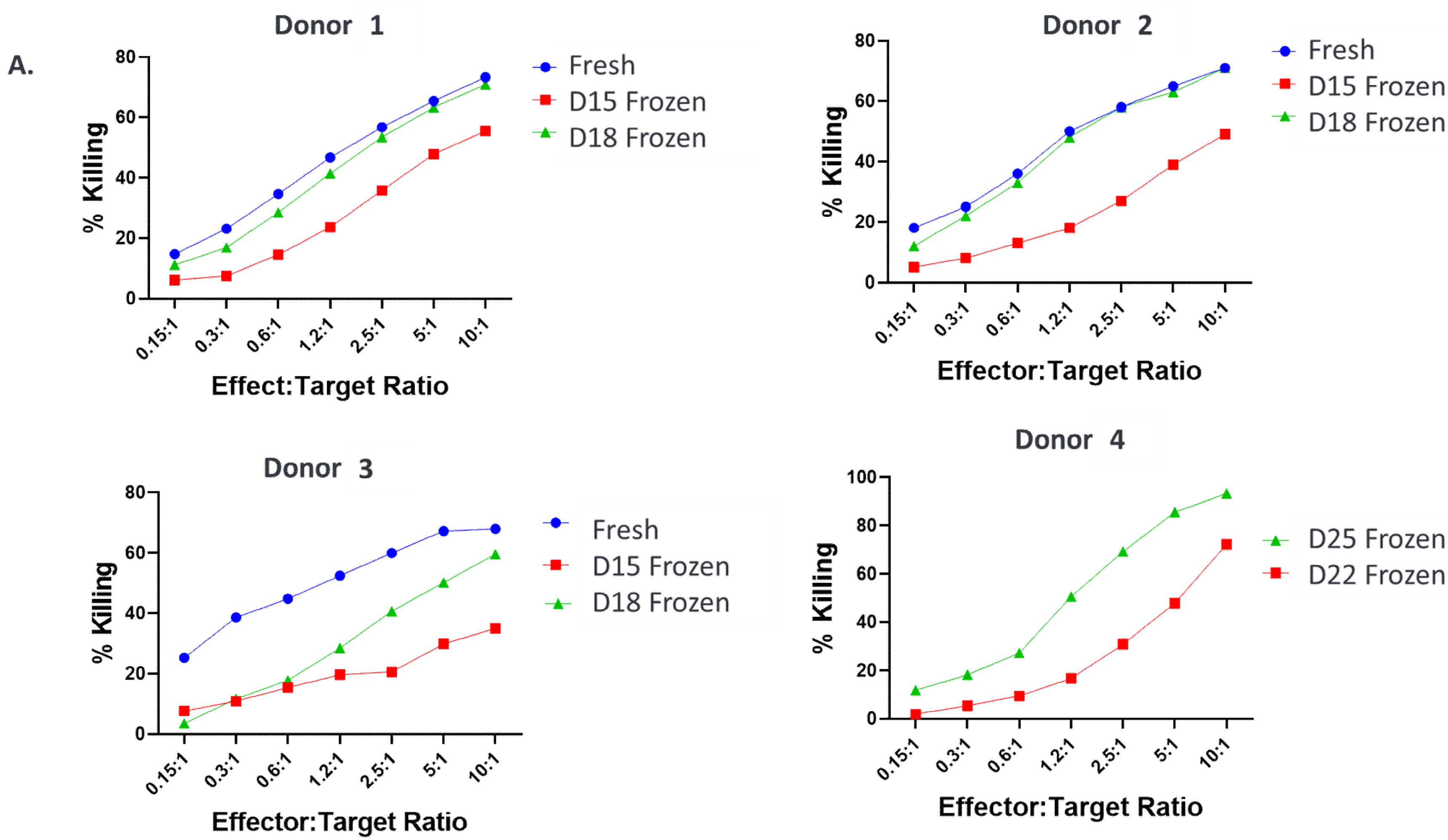

B.

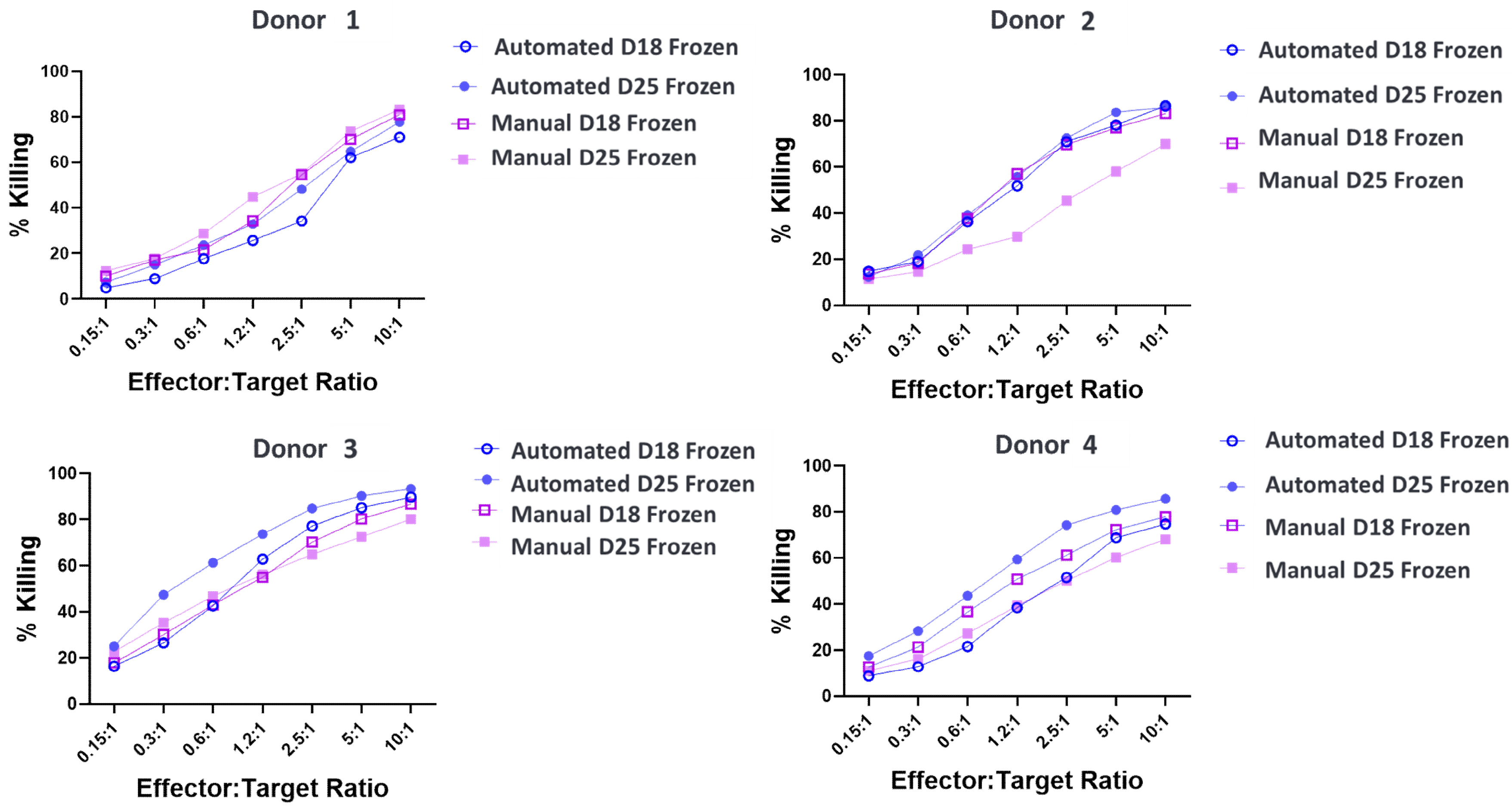
Donor 1
Donor 2
Donor 3
Donor 4
% Killing
Effector:Target Ratio
0.15:1
0.3:1
0.6:1
1.2:1
2.5:1
5:1
10:1
0
20
40
60
80
100
Automated D18 Frozen
Automated D25 Frozen
Manual D18 Frozen
Manual D25 Frozen

C.

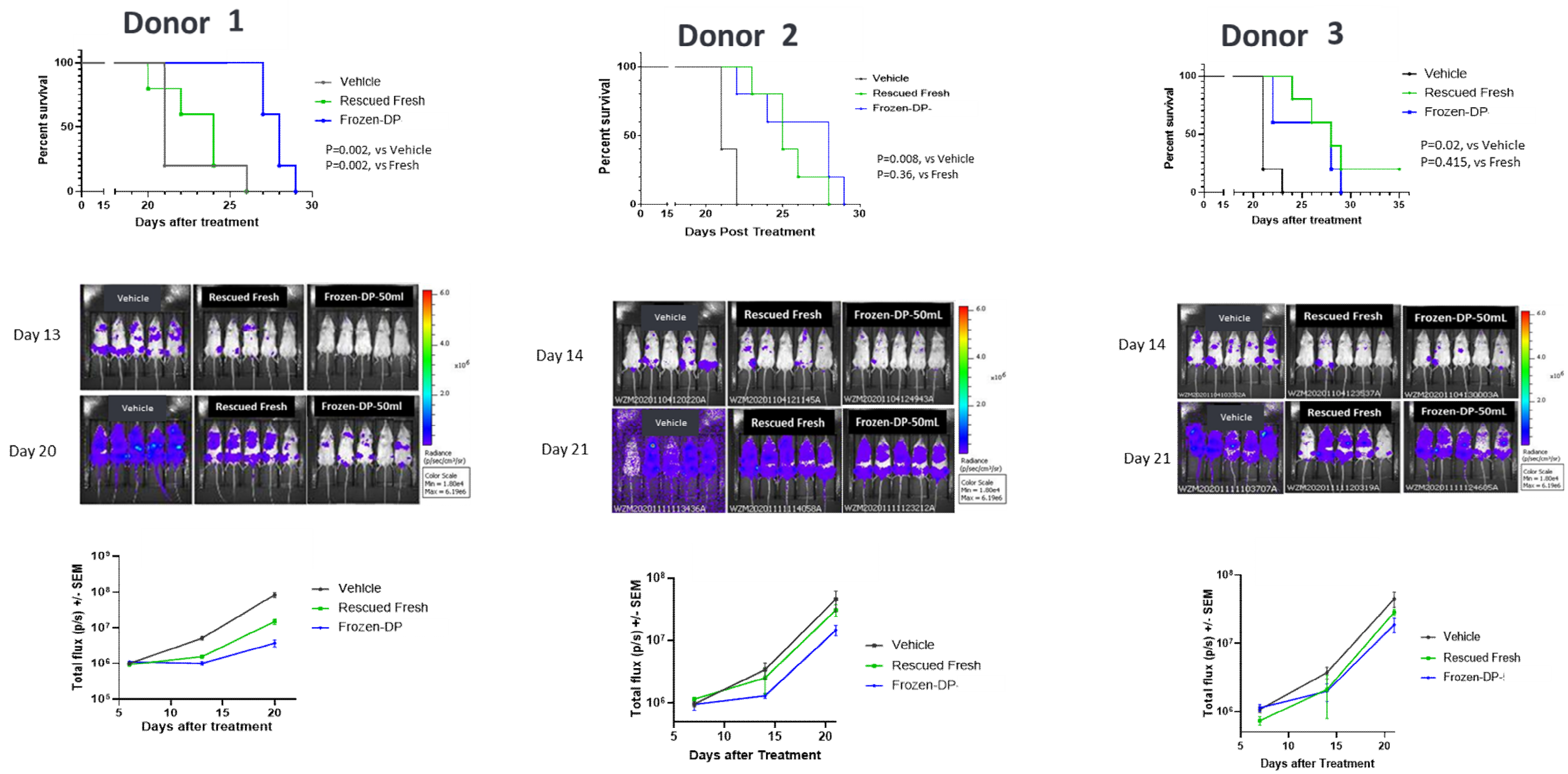
Donor 1
Donor 2
Donor 3
Percent survival
Days after treatment
Days Post Treatment
Vehicle
Rescued Fresh
Frozen-DP
P=0.002, vs Vehicle
P=0.002, vs Fresh
P=0.008, vs Vehicle
P=0.36, vs Fresh
P=0.02, vs Vehicle
P=0.415, vs Fresh
Day 13
Day 20
Day 14
Day 21
Frozen-DP-50ml
Frozen-DP-50mL
Total flux (p/s) +/- SEM
Days after Treatment

D.

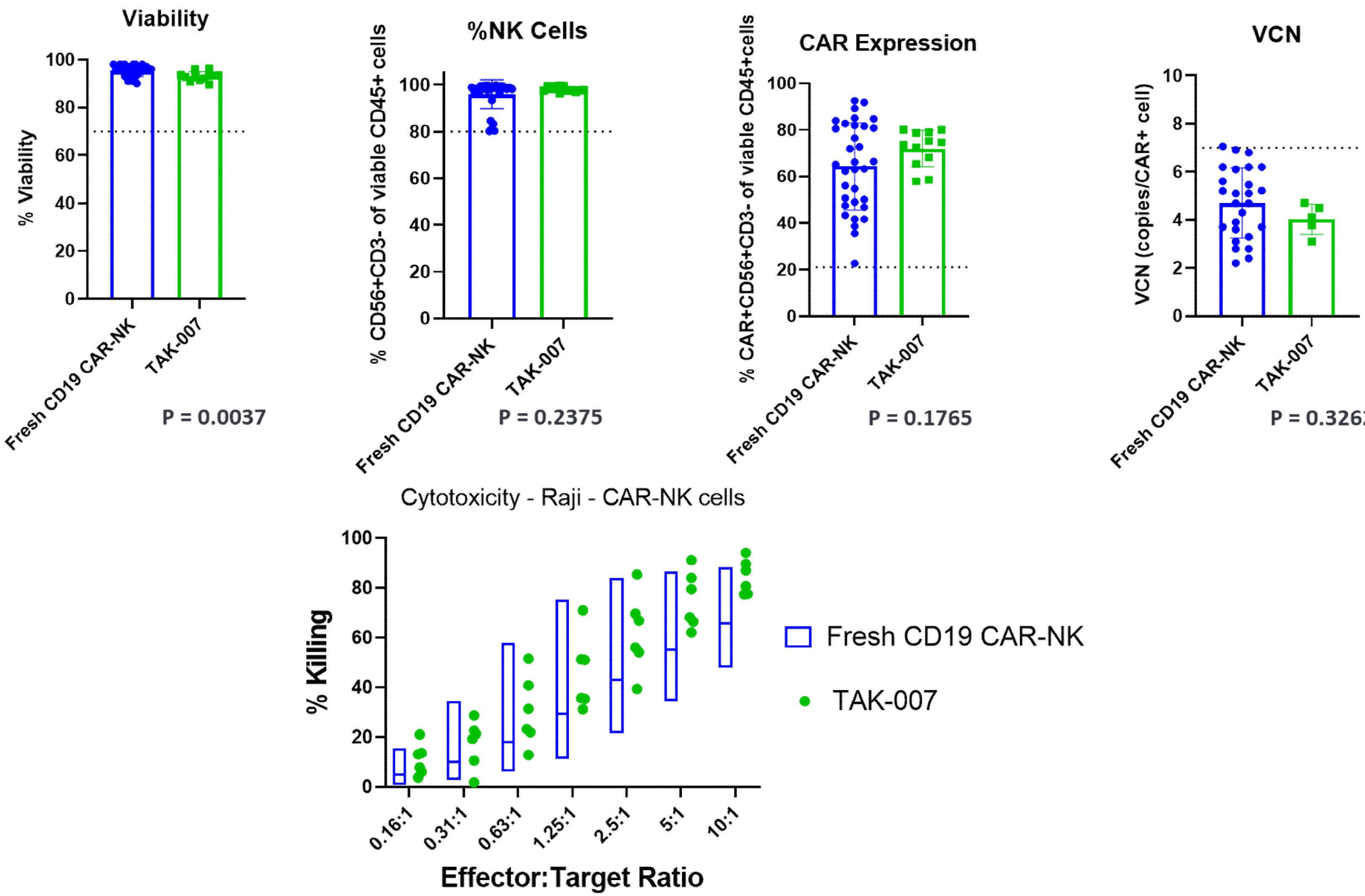

Viability
% Viability
100
80
60
40
20
0
Fresh CD19 CAR-NK
TAK-007
P = 0.0037
%NK Cells
% CD56+CD3- of viable CD45+ cells
100
80
60
40
20
0
Fresh CD19 CAR-NK
TAK-007
P = 0.2375
CAR Expression
% CAR+CD56+CD3- of viable CD45+cells
100
80
60
40
20
0
Fresh CD19 CAR-NK
TAK-007
P = 0.1765
VCN
VCN (copies/CAR+ cell)
10
8
6
4
2
0
Fresh CD19 CAR-NK
TAK-007
P = 0.3262
Cytotoxicity - Raji - CAR-NK cells
% Killing
100
80
60
40
20
0
0.16:1
0.31:1
0.63:1
1.25:1
2.5:1
5:1
10:1
Effector:Target Ratio
Fresh CD19 CAR-NK
TAK-007

A. *In vitro* cytotoxicity assessment of CD19 CAR-NK cells as fresh cell control and cryopreserved on Day 15 and Day 18. Improved cytotoxicity is demonstrated in the frozen cell product harvested on day 18 or 25 than earlier on day 15 or day 22 in 4 donors, respectively.

B. *In vitro* cytotoxicity assessment of CD19 CAR-NK cells harvested and cryopreserved on Day 18 and Day 25 with either semi-automated manufacturing process or manual manufacturing process (follow protocol operation from MDACC) respectively (1, 2). Day 25 harvested and cryopreserved cell product demonstrated similar potency compared to that of Day 18.

C. *In vivo* anti-tumor efficacy assessment of fresh cell control and CD19 CAR-NK cells manufactured with semi-automated process, harvested on Day 25 and cryopreserved in optimized cryoformulation, and optimized controlled freezing in large volume cryovials and rapid thawing process. The optimized cryoproduct of CD19 CAR-NK cells demonstrated similar survival benefit to the fresh cell control which was obtained by restimulation of frozen cell product with uAPC and then harvested and infused as fresh cells

D. Similarity analysis of fresh CD19 CAR-NK cell product and TAK-007 cryoproduct in terms of viability, NK purity, CAR%, VCN/CAR+ and cytotoxicity. 26 GMP batches of fresh CD19 CAR-NK product and 6 batches of TAK-007 were compiled for similarity analysis.

CAR, chimeric antigen receptor; D, day; GMP, Good Manufacturing Practice; NK, natural killer; uAPC, universal antigen-presenting cell; VCN, vector copy number.

References:

1. Marin D, Li Y, Basar R, Rafei H, Daher M, Dou J, et al. Safety, efficacy and determinants of response of allogeneic CD19-specific CAR-NK cells in CD19+ B cell tumors: a phase 1/2 trial. Nat Med 2024;30:772–84.
2. Liu E, Marin D, Banerjee P, Macapinlac HA, Thompson P, Basar R, et al. Use of CAR-transduced natural killer cells in CD19-positive lymphoid tumors. N Engl J Med 2020;382:545-53.
