# Supplementary material for: A Phase II, Open-label, Multicenter Study of the Safety and Efficacy of TAK-007 in Adult Patients with Relapsed/Refractory B-cell Non–Hodgkin Lymphoma
Source: Blood Cancer Discov. 2026 Apr 17;7(6):892–908. doi: 10.1158/2643-3230.BCD-25-0323 (PMC13728790; doi:10.1158/2643-3230.BCD-25-0323)
Supplement: Supplementary file 7 [file bcd-25-0323_table_s1_suppst1.pdf]

Supplementary Table 1: Representativeness of study patients

**Cancer type/subtype(s)**	B-cell Non-Hodgkin Lymphoma, including large B-cell lymphoma (LBCL) and indolent non-Hodgkin lymphoma (iNHL)
**Considerations related to:**	
**Sex**	In the US, non-Hodgkin lymphoma is more common in men (22.4 per 100,000 persons) than in women (15.7 per 100,000 persons) (1). The mortality rate is also higher in the male population (6.4% per 100,000s persons) compared to the female population (3.7% per 100,000s persons) (1).
**Age**	The median age at which patients are diagnosed with NHL in the US is 67 years, with more than half of diagnoses made in those >65 years old; the median age at death from NHL is 76 years (1, 2).
**Race/ethnicity**	In the US, white and non-Hispanic people have the highest risk of non-Hodgkin lymphoma, while Asian, Pacific Islander, American Indian, and Black populations are at lowest risk (1). The incidences per 100,000 are: White (male: 24.2% and female 16.6%), Hispanic (male 20.2% and female 15.6%), Black (17.2% male and 11.9% female), American Indian/Alaska Native (18.6% male and 11.9% female), and Asian/Pacific Islander (16.5% male and 11.7% female) (1).
**Geography**	In 2025, it is estimated that there will be 80,350 new cases and 19,390 deaths attributed to non-Hodgkin lymphoma in the US; this represents 3.9% of all new cancer cases in the US (1). Incidence rates vary globally. In 2020, the highest rates were reported by

	Israel (13.3 per 100,000), Slovenia (13.2 per 100,000), and Australia (13.1 per 100,000), compared with 12.1 per 100,000 in the US. The lowest rates were 9.6 per 100,000 in Japan, 6.4 per 100,000 in the Republic of Korea, 4.3 per 100,000 in China, and 6.4 per 100,000 in Thailand (3). The distribution by the subtype differs by geographic region (3).
**Other considerations**	NHL is a diverse disease with numerous subtypes, each with distinct characteristics (4). This study enrolled patients with relapsed/refractory NHL, including patients with LBCL or iNHL who have received ≥2 prior systemic therapies, and patients with iNHL who have failed ≥2 prior systemic therapies. The interpretation of the representativeness of this study should be approached with caution due to the small study population (*N* = 26). One patient accounted for 3.8% of the study population.
**Overall representativeness of this study**	Overall, the median age of 64.0 years in our phase 2 study is slightly lower than the median age at which patients in the US are diagnosed with NHL. The number of males vs females in our study was 69% vs 31%, which represents a slight imbalance towards the number of male patients when compared to the overall incidence rate in the US. Given the small study size, the approximate scope of this imbalance was driven by just 2 patients. All patients were enrolled at sites across the United States. Patients enrolled in our study were mostly White (88.5%), with only 1 (3.8%) patient of Black/African American origin, 1 (3.8%) patient of Asian origin and 1 (3.8%) patient of multiple origins. Most patients (92.3%) were not Hispanic or Latino in ethnicity. While the incidence rate of NHL is slightly

	higher in white individuals, and the population of the US is predominantly white (75.3%) (5), this represents a slight imbalance towards white individuals in the study. The imbalance is likely caused by the small study population, which may have been further impacted by differences in demographics across study site locations.

REFERENCES

1. Surveillance, Epidemiology, and End Results (SEER) Program. Cancer stat facts: non-Hodgkin lymphoma. National Cancer Institute. Available from: https://seer.cancer.gov/statfacts/html/nhl.html. Accessed 9 Jun 2025.

2. Thandra KC, Barsouk A, Saginala K, Padala SA, Barsouk A, Rawla P. Epidemiology of non-Hodgkin's lymphoma. Med Sci (Basel) 2021;9(1):5.

3. Luo J, Craver A, Bahl K, Stepniak L, Moore K, King J, Zhang Y, Aschebrook-Kilfoy B. Etiology of non-Hodgkin lymphoma: a review from epidemiologic studies. J Natl Cancer Cent 2022;2(4):226–34.

4. Koff JL, Chihara D, Phan A, Nastoupil LJ, Williams JN, Flowers CR. To each its own: linking the biology and epidemiology of NHL subtypes. Curr Hematol Malig Rep. 2015;10(3):244–55.

5. U.S. Census Bureau. QuickFacts: United States. U.S. Department of Commerce. https://www.census.gov/quickfacts/fact/table/US/PST045224. Accessed June 10, 2025.
