# Supplementary material for: A Phase II, Open-label, Multicenter Study of the Safety and Efficacy of TAK-007 in Adult Patients with Relapsed/Refractory B-cell Non–Hodgkin Lymphoma
Source: Blood Cancer Discov. 2026 Apr 17;7(6):892–908. doi: 10.1158/2643-3230.BCD-25-0323 (PMC13728790; doi:10.1158/2643-3230.BCD-25-0323)
Supplement: Supplementary file 8 [file bcd-25-0323_table_s2_suppst2.pdf]

Supplementary Table 2. Summary of patients with detectable anti-HLA and anti-CAR antibodies.

	Dose escalation		800M dose expansion		All patients
n (%)	200M (*n* = 3)	800M (*n* = 6)	LBCL (*n* = 10)	iNHL (*n* = 7)	(*N* = 26)
**Day -5**					
Patients with any anti-HLA antibodies result available	3	5	10	4	22
Patients with detectable anti-HLA antibodies*	0	2 (40.0)	1 (10.0)	2 (50.0)	5 (22.7)
Patients with detectable donor-specific anti-HLA antibodies*	0	0	0	1 (25.0)	1 (4.5)
Patients with any anti-CAR antibodies result available	3	5	10	6	24
Patients with detectable anti-CAR antibodies†	0	0	0	0	0
**Day -4**					
Patients with any anti-HLA antibodies result available	0	1	0	0	1
Patients with detectable anti-HLA antibodies*	0	1 (100)	0	0	1 (100)
Patients with detectable donor-specific anti-HLA antibodies*	0	0	0	0	0
Patients with any anti-CAR antibodies result available	0	0	0	0	0
Patients with detectable anti-CAR antibodies†	0	0	0	0	0
**Day 14**					
Patients with any anti-HLA antibodies result available	3	6	10	7	26
Patients with detectable anti-HLA antibodies*	0	2 (33.3)	1 (10.0)	3 (42.9)	6 (23.1)
Patients with detectable donor-specific anti-HLA antibodies*	0	0	0	2 (28.6)	2 (7.7)
Patients with any anti-CAR antibodies	3	6	10	7	26

	Dose escalation		800M dose expansion		All patients
***n* (%)**	**200M (*n* = 3)**	**800M (*n* = 6)**	**LBCL (*n* = 10)**	**iNHL (*n* = 7)**	**(*N* = 26)**
result available					
Patients with detectable anti-CAR antibodies†	0	0	0	0	0
**Day 28**					
Patients with any anti-HLA antibodies result available	2	5	8	7	22
Patients with detectable anti-HLA antibodies*	0	3 (60.0)	0	3 (42.9)	6 (27.3)
Patients with detectable donor-specific anti-HLA antibodies*	0	0	0	1 (14.3)	1 (4.5)
Patients with any anti-CAR antibodies result available	2	6	8	7	23
Patients with detectable anti-CAR antibodies†	0	0	0	0	0
**Month 2**					
Patients with any anti-HLA antibodies result available	0	3	5	7	15
Patients with detectable anti-HLA antibodies*	0	1 (33.3)	1 (20.0)	3 (42.9)	5 (33.3)
Patients with detectable donor-specific anti-HLA antibodies*	0	0	0	2 (28.6)	2 (13.3)
Patients with any anti-CAR antibodies result available	0	3	5	7	15
Patients with detectable anti-CAR antibodies†	0	0	0	0	0
**Month 3**					
Patients with any anti-HLA antibodies result available	0	2	3	6	11
Patients with detectable anti-HLA antibodies*	0	1 (50.0)	1 (33.3)	2 (33.3)	4 (36.4)
Patients with detectable donor-specific anti-HLA antibodies*	0	0	0	1 (16.7)	1 (9.1)
Patients with any anti-CAR antibodies	0	2	3	7	12

	Dose escalation		800M dose expansion		All patients
n (%)	200M (*n* = 3)	800M (*n* = 6)	LBCL (*n* = 10)	iNHL (*n* = 7)	(*N* = 26)
result available					
Patients with detectable anti-CAR antibodies†	0	0	0	0	0
**Month 4**					
Patients with any anti-HLA antibodies result available	0	2	1	4	7
Patients with detectable anti-HLA antibodies*	0	1 (50.0)	0	2 (50.0)	3 (42.9)
Patients with detectable donor-specific anti-HLA antibodies*	0	0	0	1 (25.0)	1 (14.3)
Patients with any anti-CAR antibodies result available	0	2	1	4	7
Patients with detectable anti-CAR antibodies†	0	0	0	0	0
**Month 6**					
Patients with any anti-HLA antibodies result available	0	1	1	4	6
Patients with detectable anti-HLA antibodies*	0	0	0	2 (50.0)	2 (33.3)
Patients with detectable donor-specific anti-HLA antibodies*	0	0	0	1 (25.0)	1 (16.7)
Patients with any anti-CAR antibodies result available	0	1	1	5	7
Patients with detectable anti-CAR antibodies†	0	0	0	0	0
**Month 9**					
Patients with any anti-HLA antibodies result available	0	1	0	2	3
Patients with detectable anti-HLA antibodies*	0	0	0	1 (50.0)	1 (33.3)
Patients with detectable donor-specific anti-HLA antibodies*	0	0	0	1 (50.0)	1 (33.3)
Patients with any anti-CAR antibodies	0	1	0	2	3

	Dose escalation		800M dose expansion		All patients
***n* (%)**	**200M (*n* = 3)**	**800M (*n* = 6)**	**LBCL (*n* = 10)**	**iNHL (*n* = 7)**	**(*N* = 26)**
result available					
Patients with detectable anti-CAR antibodies†	0	0	0	0	0
**Month 12**					
Patients with any anti-HLA antibodies result available	0	1	0	1	2
Patients with detectable anti-HLA antibodies*	0	1 (100)	0	0	1 (50.0)
Patients with detectable donor-specific anti-HLA antibodies*	0	1 (100)	0	0	1 (50.0)
Patients with any anti-CAR antibodies result available	0	1	0	1	2
Patients with detectable anti-CAR antibodies†	0	0	0	0	0

*Percentages were based on the total number of patients with any result available for anti-HLA antibodies at each visit; †Percentages were based on the total number of patients with any result available for anti-CAR antibodies at each visit;

CAR, chimeric antigen receptor; CAR-T, chimeric antigen receptor T-cell; HLA, human leukocyte antigen; iNHL, indolent non-Hodgkin Lymphoma; LBCL, large B-cell lymphoma.
