# Supplementary material for: A Phase II, Open-label, Multicenter Study of the Safety and Efficacy of TAK-007 in Adult Patients with Relapsed/Refractory B-cell Non–Hodgkin Lymphoma
Source: Blood Cancer Discov. 2026 Apr 17;7(6):892–908. doi: 10.1158/2643-3230.BCD-25-0323 (PMC13728790; doi:10.1158/2643-3230.BCD-25-0323)
Supplement: Supplementary file 9 [file bcd-25-0323_table_s3_suppst3.pdf]

Supplementary Table 3. Categorization of drug product lots based on clinical responses*.

Drug product	Cohort	Best overall response	Eliciting a response? (Yes/No)
	Escalation	Progressive disease	
DP1	Escalation	Progressive disease	No
DP2	Escalation	Progressive disease	No
	Expansion	Progressive disease	
	Expansion	Stable disease	
	Expansion	Stable disease	
DP3	Expansion	Complete response	Yes
	Expansion	Progressive disease	
	Expansion	Progressive disease	
	Expansion	Partial response	
	Expansion	Partial response	
	Escalation	Complete response	
DP4	Expansion	Complete response	Yes
	Escalation	Partial response	
	Expansion	Partial response	
	Expansion	Partial response	
	Expansion	Complete response	
	Expansion	Complete response	
DP5	Expansion	Complete response	Yes
	Expansion	Partial response	Yes
DP6	Escalation	Complete response	
	Expansion	Complete response	

*Drug products were categorized as responsive (eliciting a clinical response) or non-responsive (not eliciting a clinical response). DP1 and DP2are non-responsive drug products, while DP3, DP4, DP5 and DP6 are responsive drug products.
